# On the prediction of non-CG DNA methylation using machine learning

**DOI:** 10.1093/nargab/lqad045

**Published:** 2023-05-17

**Authors:** Saleh Sereshki, Nathan Lee, Michalis Omirou, Dionysia Fasoula, Stefano Lonardi

**Affiliations:** Department of Computer Science and Engineering, University of California, Riverside, CA 92521, USA; Department of Computer Science and Engineering, University of California, Riverside, CA 92521, USA; Department of Agrobiotechnology, Agricultural Microbiology Laboratory, Agricultural Research Institute, Nicosia 1516, Cyprus; Department of Plant Breeding, Agricultural Research Institute, Nicosia 1516, Cyprus; Department of Computer Science and Engineering, University of California, Riverside, CA 92521, USA

## Abstract

DNA methylation can be detected and measured using sequencing instruments after sodium bisulfite conversion, but experiments can be expensive for large eukaryotic genomes. Sequencing nonuniformity and mapping biases can leave parts of the genome with low or no coverage, thus hampering the ability of obtaining DNA methylation levels for all cytosines. To address these limitations, several computational methods have been proposed that can predict DNA methylation from the DNA sequence around the cytosine or from the methylation level of nearby cytosines. However, most of these methods are entirely focused on CG methylation in humans and other mammals. In this work, we study, for the first time, the problem of predicting cytosine methylation for CG, CHG and CHH contexts on six plant species, either from the DNA primary sequence around the cytosine or from the methylation levels of neighboring cytosines. In this framework, we also study the cross-species prediction problem and the cross-context prediction problem (within the same species). Finally, we show that providing gene and repeat annotations allows existing classifiers to significantly improve their prediction accuracy. We introduce a new classifier called AMPS (annotation-based methylation prediction from sequence) that takes advantage of genomic annotations to achieve higher accuracy.

## INTRODUCTION

DNA methylation is an epigenetic mark that plays a critical role in regulating a variety of cellular processes, such as gene expression, genome stability, transposon silencing and gene imprinting [see e.g. (1–4)]. The most common type of DNA methylation is the addition of a methyl group to the fifth carbon of a cytosine residue, indicated as 5mC. In mammals, DNA methylation is mostly found at cytosines that are followed by guanine base, known as CG methylation. Long stretches of DNA that are very rich in the dinucleotide CG, called CpG islands, tend to be less methylated than the other cytosines in the genome (5–7). As said, DNA methylation is one of several epigenetic mechanisms that cells use to regulate gene expression (8,9). In humans, the dysregulation of DNA methylation is associated with a variety of diseases, including cancer (10,11) and neurological disorders (12,13). In plants and other non-vertebrates, however, cytosine methylation in the CHH and CHG contexts (where H represents any base except G) is almost as common as methylation in the CG context (14–16). It is now well understood that distinct molecular mechanisms in the cells regulate cytosine methylation and demethylation depending on the context (17–20).

Recent studies suggest the importance of non-CG methylation in both vertebrates and non-vertebrates. In humans, non-CG methylation is the most abundant form of DNA methylation in neurons and plays a critical role in cognitive functions [see e.g. (21–23)]. Dysregulation of this type of methylation has been associated with mental diseases such as schizophrenia (24). In plants, it has been shown that (i) distinct pathways and molecular processes maintain cytosine methylation in CG, CHG and CHH contexts [see e.g. (17,25)]; (ii) methylation patterns in gene body and repetitive elements differ for CG and non-CG methylation [see e.g. (25–29)]; (iii) both CG and CHG methylation are correlated to genome size and repetitive content, while CHH methylation is not (16); and (iv) methylation inheritable patterns in symmetric contexts (CG and CHG) are different from those in the nonsymmetric (CHH) context (30).

Several methods are available for reading the methylation status of cytosines. Whole genome bisulfite sequencing (also known as BS-Seq) is arguably the most common method. Other techniques include bead chip arrays (e.g. Illumina Infinium), Oxford Nanopore (31) or affinity enrichment-based techniques, such as methylcytosine-specific antibodies (MeDIP-Seq). BS-Seq allows for quantitative cytosine methylation detection at single-base resolution and is still considered the ‘gold standard’ for the analysis of DNA methylation. By treating DNA with sodium bisulfite, unmethylated cytosines transform to uracils, while methylated cytosines stay intact. Once the DNA is converted, DNA sequencing (typically carried out on Illumina instruments) generates the reads that are then mapped to the reference genome using conversion-aware mapping tools [e.g. Bismark (32), BS Seeker (33) or BRAT-nova (34)]. Since the methylation level for each cytosine is obtained by computing the ratio between the number of reads that indicate a methylated cytosine and the number of mapped reads, the statistical confidence associated with this measurement depends on the depth of sequencing coverage at each cytosine and the bisulfite conversion rate. To guarantee that the read coverage is sufficient for all the cytosines in the genome, the average sequencing depth needs to be high, which can be expensive for large eukaryotic genomes. Since sequencing depth is not uniform across the genome, some cytosines can end up with low or no read coverage, which prevents the accurate measurement of their methylation level. This problem is particularly acute for single-cell experiments because the coverage is usually much lower and less uniform than bulk sequencing data.

As a result, several methods have been developed in the last 10 years for predicting or imputing cytosine methylation levels. These methods are mostly focused on prediction of CG methylation in humans. In one of these studies, a deep neural network used sequence and methylation level of neighboring cytosines to predict the methylation level from single-cell experiments, exclusively for the CG context in human and mouse cells (35). Another method targeted at CG methylation prediction on mouse single-cell data was proposed by Li and Liu (36) using again deep learning. Their model uses the underlying DNA sequence, the methylation status and the distance of neighboring cytosines to carry out methylation prediction. Both Tian *et al.* (37) and Zeng and Gifford (38) used a convolutional neural network to predict CG methylation levels from the DNA sequence in the human genome. De Waele *et al.* (39) used a transformer architecture for imputation of single-cell methylation levels in humans and mice. In (40), a large language model based on BERT transformers was used to predict cytosine methylation, cytosine hydroxymethylation and adenine methylation from the primary DNA sequence. Wang *et al.* (41) proposed a CNN for predicting histone marks H3K4me3, H3K27me3 and H3K9ac, cytosine DNA methylation, adenine DNA methylation and adenine RNA methylation from the primary sequence.

Other studies used Illumina Infinium Human Methylation 450 array data to carry out predictions. In one of these studies, Zhang *et al.* (42) proposed a random forest (RF) model that uses the DNA sequence, the neighboring cytosines’ methylation levels and the presence of CpG islands to predict CG methylation levels in humans. In a similar study, Zheng *et al.* (43) used an RF model to predict cytosine methylation levels in humans from Infinium methylation levels and the distance of neighboring CG.

All these studies demonstrate that it is possible to predict CG methylation from the DNA sequence or the neighboring methylation levels at various levels of accuracy. However, the problem of predicting non-CG methylation has been so far largely ignored despite its growing importance in molecular biology. Even worse, sometimes non-CG methylation is improperly bundled with CG prediction, despite clear mechanistic differences at the cellular level. Here we address for the first time, to the best of our knowledge, this fundamental shortcoming. Specifically, our work makes the following contributions: (i) We study the problem of predicting cytosine methylation independently for the CG, CHG and CHH contexts (and for all three contexts mixed) on six plant species on either the DNA primary sequence or the methylation level of neighboring cytosines. (ii) We study the cross-context prediction problem; i.e. we investigate how hard it is to predict methylation for a specific context when trained on a different one. (iii) We study the cross-species prediction problem; i.e. we investigate how hard it is to predict methylation for a specific species when trained on a different one. (iv) We show that one can obtain higher predictive accuracy from the levels of neighboring cytosines than from the DNA sequence. (v) We show that providing gene and repeat annotations allows any classifier to significantly improve its prediction accuracy. (vi) We introduce a new classifier called AMPS (annotation-based methylation prediction from sequence) that outperforms state-of-the-art methylation predictors by taking full advantage of the annotations. (vii) We identify a set of statistically significant motifs that contribute to context-specific DNA methylation in the species included in this study.

## MATERIALS AND METHODS

### Data sources and data pre-processing

BS-Seq data for *Arabidopsis thaliana*, rice (*Oryza sativa*), tomato (*Solanum lycopersicum*), cucumber (*Cucumis sativus*) and marchantia (*Marchantia polymorpha*) were obtained from the Sequence Read Archive (SRA) of NCBI/NIH. BS-Seq data for cowpea (*Vigna unguiculata*) were generated in the context of the Cyprus national project ‘Cowpea breeding and adaptation to climate change’ (44). The cowpea genome was recently sequenced and assembled by our group (45). The other genomes were obtained from NCBI (see Supplementary Table S1 for source and assembly versions).

Supplementary Figure S20 shows the location of these six species on a phylogenetic tree of the major land plant species (46). These plant species belong to six distinct orders: *Arabidopsis* belongs to the Brassicales, cowpea to the Fabales, cucumber to the Cucurbitales, tomato to the Solanales, rice to the Poales and marchantia to the Marchantiales. Not all plant orders are represented in our study, but we plan to expand it to the other orders in the future.

Read quality was checked using FastQC v0.11.5. In some cases, sequencing primers were detected in the sequenced reads. Reads that had these anomalies were trimmed with Trimmomatic v0.33 (47). Reads were mapped against the corresponding reference genome using Bismark v0.22.2 using default parameters (32). Only reads that were uniquely aligned were used by Bismark; i.e. ambiguous reads with multiple mappings were discarded.

The output of Bismark was processed using custom scripts as follows. First, the methylation level of each cytosine was obtained by computing the ratio of the number of methylated reads over all the reads covering that cytosine. A cytosine was declared to be *methylated* if the methylation level was at least 0.5, *unmethylated* otherwise. We are aware that this threshold might be too strict for non-CG methylation (in particular for CHH), but we had to be consistent with the 50% threshold used in MRCNN (37), CpGenie (38) and the RF classifier (42). Methylation was called only for cytosines that had a coverage of >10 reads. Cytosines covered by <11 reads had an unknown methylation status and were not used for training or testing.

### Gene body methylation profiles

To obtain the average species-specific gene methylation profile, we collected the methylation levels for each annotated gene, as well as the methylation levels in 2 kb upstream and downstream of each gene. Gene bodies and flanking regions were split into 5 bins each, for a total of 15 bins. For each bin *r* ∈ [1, 15], the average methylation level *M*(*r*) was calculated as follows:


$$\begin{eqnarray*} M(r) = \left(\sum _{i=1}^{G} m(r,i)l(r,i) \right) / \left( \sum _{i=1}^{G}{l(r,i)} \right) ,\end{eqnarray*}$$

where *G* is the total number of annotated genes in that species, *m*(*r*, *i*) is the ratio of methylated cytosines over all cytosines in bin *r* of gene *i* and *l*(*r*, *i*) is the length of bin *r* in gene *i* (a bin is 400 bp for flanking regions; it is one-fifth of a gene for the bins within a gene).

### Training set design

For classifiers that rely on the DNA sequence, a context-specific training set was composed of *n* DNA sequences of length *W*_*s*_ centered at a cytosine (i.e. *W*_*s*_/2 bases upstream and *W*_*s*_/2 bases downstream of the cytosine) chosen uniformly at random among all possible cytosines that belonged to that particular context (either CG, CHH or CHG), in which *n*/2 were methylated (i.e. have a methylation level of at least 0.5) and *n*/2 were unmethylated (i.e. have a methylation level below 0.5). The training set was balanced because the highly skewed distribution in some contexts could make the prediction trivial. For example, >99% of cytosines in the CHH context for *Arabidopsis* are unmethylated; thus, a ‘classifier’ that predicts every cytosine in the CHH context for *Arabidopsis* to be unmethylated would achieve >99% accuracy. In contrast, almost 90% of cytosines in the CG context for tomato are methylated; thus, a ‘classifier’ that predicts every cytosine in the CG context for tomato to be methylated would achieve almost 90% accuracy. In some cases, *n* was limited by the number of available methylated cytosines genome-wide (e.g. CHH in *Arabidopsis*; see Supplementary Table S4). Even in those cases, however, we kept the training set balanced in terms of methylated/unmethylated cytosines. For the combined context (indicated as ‘ALL’ in the figures), we balanced the three contexts (CG, CHG and CHH) in equal proportions because otherwise the skewed distribution in some contexts could make the prediction trivial. For example, for tomato a classifier that calls (i) all cytosines in the CG context methylated, (ii) all cytosines in the CHG context methylated and (iii) all cytosines in the CHH context unmethylated would achieve an expected 92% accuracy, based on Supplementary Table S6 and some basic probability calculations (not shown). Since the DNA sequences were one-hot encoded, the training set was composed of *n* binary matrices of size *W*_*s*_ × 4. Several choices of the window size *W*_s_ and the training set size were tested, as explained in the ‘Effect of the window size and training set size on the prediction accuracy’ section.

For classifiers that rely on genomic annotations (in addition to the primary DNA sequence), the one-hot encoded *W*_*s*_ × 4 input was augmented with a few bit vectors representing the annotations. We used two bit vectors to represent gene annotations (one for each strand) and one bit vector for the repeats. The binary values of these bit vectors indicated the annotation status of each nucleotide in the window. If a nucleotide was contained in a particular functional element (e.g. coding sequence), the corresponding value in the strand-specific bit vector was 1 (and zero otherwise). Supplementary Table S5 lists the functional elements used for each species.

Repeat annotations for marchantia were downloaded from PlantRep (48). For the other species in this study, RepeatMasker v4.1.2 was used to annotate the genome for repeats. The default repeat database was used for *Arabidopsis*, rice and tomato. The repeat library for cucumber was downloaded from msRepDB (49). The repeat library for *Phaseolus vulgaris* was used for cowpea.

For classifiers that rely on the methylation level of neighboring cytosines, the context-specific training set was composed of *n* vectors of length *W*_*p*_, where the first *W*_*p*_/2 components of the vector are methylation levels (in the range [0, 1]) of the cytosines upstream and the second *W*_*p*_/2 components of the vector are methylation levels of cytosines downstream of a cytosine chosen uniformly at random among all possible cytosines that belong to that particular context (either CG, CHH or CHG). For the combined context, the training set was composed of an equal number of examples from CG, CHG and CHH. Again, we made sure that the training set was balanced: *n*/2 samples had a center cytosine that was methylated and *n*/2 samples had an unmethylated center cytosine. Please note that while the center cytosine is context-specific, the vector contained methylation levels for cytosines in any context, as long they had sufficient read coverage (i.e. >10 reads).

In all experiments, 80% of the data was used for training, 10% was used for validation and 10% was used for testing. Validation and test data sets had the same characteristics of the training set, but we made sure no DNA sequence in the training set appeared in the test set.

### Classifiers

We first studied the prediction accuracy of an RF because RF has been used in the literature for this problem [see e.g. (42,43)]. RF was implemented using Python Scikit-learn (version 0.24.2) and trained with 50 estimators and unlimited tree depth. All other parameters for RF were the defaults in the Scikit-learn module.

The most effective ML methods in the literature to predict cytosine methylation are, however, based on deep learning [see e.g. (35–39)]. To carry out an extensive set of prediction experiments, we created a deep learning architecture based on CNNs.

We called our architecture for the prediction of cytosine methylation AMPS. As explained in the previous section, the input to AMPS is a matrix of size *W*_*s*_ × (4 + *a*), where *a* is the number of bit vectors representing the annotations (*a* = 0 when AMPS uses only the sequence, i.e. no annotations). The input was first processed by a 1D convolutional layer with kernel size of (4 + *a*). This convolution layer had 16 channels followed by a ReLU function. The next layer was a fully connected layer with 128 nodes using a ReLU activation function. To avoid overfitting, a dropout ratio of 0.5 was used for the fully connected layer. The last layer was a single node using a sigmoid activation function. A stochastic gradient descent optimizer was used, and the loss function was binary cross-entropy. The architecture of AMPS is illustrated in Supplementary Figure S2.

The input to the network for the prediction of cytosine methylation from neighboring cytosines was a vector of methylation levels in the range [0, 1] of length *W*_*p*_. The network was composed of four fully connected layers with 20, 16, 8 and 1 node, respectively. The hidden layers used ReLU as their activation function. A dropout ratio of 0.5 was used to prevent overfitting. A stochastic gradient descent was used for optimization, and binary cross-entropy was used for the loss function.

We also designed a CNN-based architecture that predicts cytosine methylation from the (i) sequence, (ii) annotation and (iii) methylation levels of neighboring cytosines. The DNA sequence and the annotation were provided as a matrix of size *W*_*s*_ × (4 + *a*), where *a* is the number of annotations. This portion of the input was processed through two convolutional layers followed by a ReLU activation function and a flatten layer. The resulting vector was combined with a vector of length *W*_*p*_ for the methylation levels of *p* neighboring cytosines. The combined vector was processed through three fully connected layers with 16, 8 and 1 node, respectively. The first two layers were followed by a ReLU activation function, while the last one was processed by a sigmoid activation function. A dropout rate of 0.5 was used in the fully connected layers to prevent overfitting. A stochastic gradient descent was used for optimization, and binary cross-entropy was used for the loss function. The architecture was trained with batch size 32, 20 epochs and a learning rate of 0.001.

### Motif finding

We used Grad-CAM to score the importance of the input position for the prediction of the methylation status. Grad-CAM is a tool for the analysis of CNN architectures to determine the importance of pixels in an image to determine the correct label (50). After training AMPS for a specific species and context, we selected 10 000 inputs that were correctly classified as methylated and 10 000 inputs that were correctly classified as unmethylated. The two sets were given in input to Grad-CAM (along with the weights of the AMPS network) to score the importance of each position in the input vectors. Since the input has 3200 dimensions, we selected the most important subsequence by sliding a window of length 50 along the input and reporting the window with the highest average. The DNA sequences corresponding to those windows were fed into MEME v5.4.1 (51), using default parameters. The top 10 motifs produced by MEME were recorded for each species and each context, separately for methylated and nonmethylated inputs. The top motifs were matched against the plant motif database JASPAR 2020 (52) using TOMTOM v5.4.1 (51).

## RESULTS

### Context- and species-specific prediction

As said earlier, while vertebrate DNA cytosine methylation is primarily found in the CG context, plants have significant levels of DNA methylation in the CG, CHG and CHH contexts (17,53). To investigate CHG and CHH methylation, we selected six plant species, namely (i) *A. thaliana* representing the Brassicales order, (ii) rice (*O. sativa*) representing the Poales order (the only monocotyledons in our study), (iii) tomato (*S. lycopersicum*) representing the Solanales order, (iv) cucumber (*C. sativus*) representing the Cucurbitales order, (v) cowpea (*V. unguiculata*) representing the Fabales order and (vi) the early land plant *M. polymorpha* representing the Marchantiales order (the only non-angiosperm in this study). We selected these species to cover a wide range in the phylogenetic tree of the plant kingdom (see Supplementary Figure S20), including a non-angiosperm. Data sources and the processing of BS-Seq reads are described in the ‘Materials and Methods’ section. Supplementary Table S2 summarizes the main statistics of the BS-Seq reads for each plant species.

The average cytosine coverage from BS-Seq mapped reads ranged from 5× in tomato to 21× in *Arabidopsis* (see Supplementary Table S3). To ensure high statistical confidence in the determination of methylation levels, a strict threshold for coverage was adopted; we only called cytosines that were covered by >10 reads. A cytosine was considered methylated if more than half of the reads covering it indicated methylation (and unmethylated otherwise). We are aware that this threshold might be too strict for non-CG methylation (in particular for CHH), but we had to be consistent with the 50% threshold used in MRCNN (37), CpGenie (38) and the RF classifier (42). It is well known that different contexts exhibit differences in average per base cytosine methylation. Observe that the percentage of CG, CHG and CHH methylation varies greatly among different species. Supplementary Table S4 shows that >89% of cytosines in the CG context are methylated in tomato compared to only ∼27% in *Arabidopsis*, >62% of cytosines in the CHG context are methylated in tomato compared to only 11% in *Arabidopsis* and 2.73% of cytosines in the CHH context are methylated in tomato compared to 0.17% in marchantia.

Our cytosine prediction analyses can be logically organized in six steps, which are described hereafter and summarized in Supplementary Figure S1.

**Figure 1.**
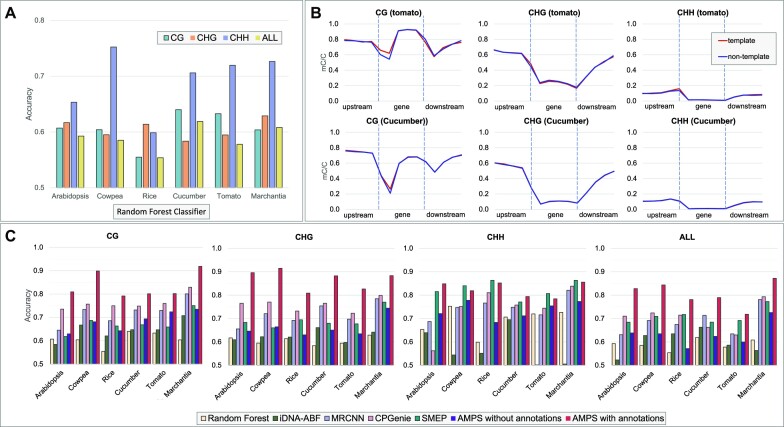
(**A**) Context-specific species-specific prediction accuracy of an RF binary classifier on the six plant species included in this study. (**B**) Context-specific gene body methylation levels for tomato (top row) and cucumber (bottom row) for the template and nontemplate strands. (**C**) Context-specific species-specific prediction accuracy for RF, iDNA-ABF, MRCNN, CpGenie, SMEP, AMPS without annotations and AMPS with annotations (AMPS is the new method proposed here).

In the first step, we established a baseline for the methylation classification problem using a simple classifier, i.e. RF. Figure 1A shows the accuracy of RF on *Arabidopsis*, cowpea, rice, cucumber, tomato and marchantia independently for each context (CG, CHG, CHH) and for all contexts mixed (ALL). First, observe that the prediction performance of cytosine methylation from the sequence is highly dependent on the context. Also observe that the prediction of methylation in the CHH context is often more accurate than the prediction of methylation in the other two contexts, which suggests that the CHH methylation could be more sequence dependent in plants than CG or CHG. Mixing all the contexts results in a decrease in classification performance, which supports the need of an individual classifier for each context.

In the second step, we investigated the observations reported in the literature that the methylation levels in plants vary drastically in gene bodies compared to upstream and downstream regions (25–28). Figure 1B shows the methylation levels for template and nontemplate strands in the gene body and 2 kb flanking regions averaged over all genes in tomato and cucumber (see the ‘Materials and Methods’ section for details). Supplementary Figure S3 shows the corresponding analyses for the other plant species in this study. Observe that CG and CHG methylation levels dip in correspondence to the gene boundaries, and that overall shape of methylation levels is context dependent. Similarly, it has been shown that the methylation patterns in plants are dramatically different in repetitive regions of the genome compared to the nonrepetitive regions. Methylation levels for all contexts are the highest in repetitive regions, mainly as a means to silence transposable elements (16,54).

These analyses prompted the question of whether providing the classifier with genomic annotation information (e.g. gene boundaries, coding sequence boundaries, intron/exon boundaries and repeats) could boost the classification performance for cytosine methylation. To answer this question, we designed a new classifier that uses the annotations listed above in addition to the DNA sequence. Our classifier, called AMPS, is a deep learning architecture that uses convolutional neural networks (see details in the ‘Materials and Methods’ section). Since we planned to use annotations related to genes and repeats, we investigated how much of each genome is annotated by these genomic features. Supplementary Figure S18 shows that the 65% of the smallest genome (*Arabidopsis*) is annotated as a gene, while only 17% of the largest genome (tomato) is annotated as a gene. The fraction of each genome annotated as repetitive ranges from 16% (*Arabidopsis*) to 43% (cowpea). Supplementary Figure S19 shows the context-specific species-specific fraction of all cytosines covered by annotations. To determine whether annotations would improve the classification accuracy, we carried out a comparative analysis against previously published methods, as well as our classifier without annotations, which led to the third step in the analysis.

In the third step, we compared the performance of AMPS to RF, CpGenie (38), MRCNN (37), iDNA-ABF (40) and SMEP (41). We chose to compare AMPS against CpGenie, MRCNN, iDNA-ABF and SMEP because they are considered state-of-the-art methods for methylation prediction exclusively from DNA sequence. In fairness, we should note that most of these tools were optimized for predicting methylation in the CG context on the human genome. We retrained all these tools on our species-specific and context-specific plant data set, but their architectures might not be optimal for non-CG nonhuman methylation. We should also note that most of these tools use a more sophisticated deep learning architecture than AMPS, resulting in a larger number of weights and hyperparameters. iDNA-ABF converts the input DNA sequence into *k*-mers and then feeds them into a BERT encoder. iDNA-ABF was trained using a learning rate of 0.000005 and batch size of 256 because the default parameters prevented us to retrain it on our plant data sets. SMEP is a CNN-based architecture that was retrained using the parameters provided by the authors. The hyperparameters of AMPS were not highly optimized to ensure that the method would be able to generalize, but the effect of window size and the training set size on the prediction performance was extensively studied in the ‘Effect of the window size and training set size on the prediction accuracy’ section. In all experiments, AMPS’ window size (with or without annotation) was 3.2 kb, CpGenie’s window size was 1 kb, MRCNN’s window size was 400 bp, iDNA-ABF’s window size was 71 bp and SMEP’s window size was 41 bp. These window sizes were prescribed by the corresponding architectures proposed by the authors. All classifiers were trained on 500 000 DNA sequences selected uniformly at random from the genome (a discussion about training set size can be found in the ‘Materials and Methods’ section), if available. As explained in the ‘Effect of the window size and training set size on the prediction accuracy’ section (and shown in Supplementary Figure S7), the variance in performance across multiple random samples was negligible, so all the experiments were carried out on a single sample to reduce the overall computational cost.

Figure 1C reports the accuracy of the classifiers listed above, including AMPS without annotations. Observe that (i) in the CG, CHG and ALL contexts, AMPS (with annotations) achieved higher accuracy than the other five methods on all six species (SMEP performed better than AMPS in the CHH context on four species out of six), (ii) AMPS with annotations had the biggest improvement over AMPS without annotations in the CHG context (which is the context in Supplementary Figure S19 that has the highest percentage of cytosines covered by gene annotations, irrespective on the species) and (iii) in some cases, the accuracy of AMPS without annotation was lower than other predictors, suggesting the critical advantage of using genomic annotation as an input feature. Also, observe in Figure 1C that (i) the accuracy of different classifiers is context dependent and (ii) in 23 out of 30 experiments, the prediction accuracy that used all the contexts mixed was lower than training on each context independently. The same experimental results are shown in Supplementary Figure S8, but grouped by classifier instead of species. Supplementary Figure S9 compares the performance of AMPS when using different types of annotations (no annotation, only repeats, only genes or repeats + genes). Observe that gene annotations helped more than repeat annotations in 19 of the 24 experiments. AMPS’ performance when using both repeats and gene annotation was always the best. To determine which gene annotation was the most informative, we measured the accuracy of AMPS on *Arabidopsis* using individual functional element, namely gene, CDS or exons. Supplementary Figure S12 shows that each annotation by itself performs as well as all functional annotations combined. We wondered whether the performance of MRCNN, RF and CpGenie could be rescued if they had used functional annotations. For this purpose, we modified the input layer of RF, MRCNN and CpGenie to allow sequence and annotations as input. Supplementary Figure S11 shows that in all cases the prediction accuracy for MRCNN and AMPS improved using annotations. For RF, the prediction accuracy improved in 22 out of 24 cases. For CpGenie, the prediction accuracy improved in 23 out of 24 cases. Observe that (i) often the improvement in prediction accuracy was very significant and (ii) the only three cases in which the annotation degraded the performance are for CHH. Finally, Supplementary Figure S10 compares the performance of the four methods when annotations are used. Observe that in 16 out of 30 experiments AMPS achieved the highest accuracy.

While accuracy is the main metric of performance for classifiers trained on balanced data sets, other statistical measures can be considered for choosing the best classifier. In Supplementary Figure S4, we report precision, recall and *F*1 score for all contexts and all species for the tools listed in Figure 1C. Observe that (i) in 17 out of 24 experiments, AMPS (with annotation) achieved a higher precision than the other tools, (ii) in 17 out of 24 experiments, AMPS (with annotation) achieved a higher recall than the other tools, and (iii) in 20 out of 24 experiments, AMPS (with annotation) achieved a higher *F*1 score than the other tools.

### Cross-context and cross-species prediction

**Figure 2.**
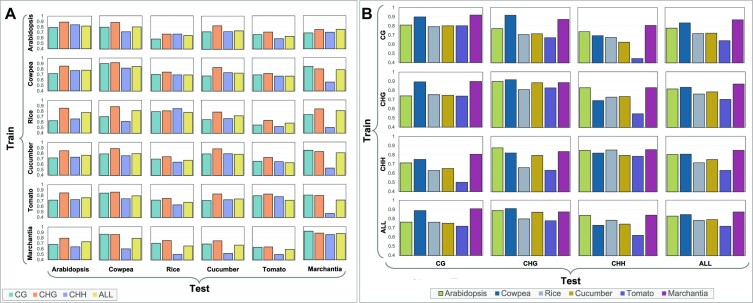
(**A**) Cross-species methylation prediction accuracy of AMPS with annotation (for the subset of annotations shared by all the species). (**B**) Cross-context methylation prediction accuracy of AMPS with annotations.

In the fourth step, we investigated the ability of the predictor to carry out cross-species prediction from the DNA sequence and annotations. Figure 2A shows the accuracy of AMPS with annotations when trained with one species and tested on another, for each context individually and all contexts mixed. In this case, we could not use all annotations because of the different number of functional elements available for each organism, so we used only the subset of annotations shared by all the species. Observe that training and testing on the same species achieves the highest accuracy, as expected. Supplementary Figure S13 shows the performance of AMPS without annotation when trained on one species and tested on another. Again, the highest accuracy was obtained when training and testing on the same species.

In the fifth step, we investigated cross-context predictions. The performance of AMPS (with annotations) was evaluated when trained on one context and tested on another. Figure 2B shows the prediction accuracy for all pairs of training/testing contexts (including the mixed contexts) for all species. Observe that in most of the cases, cross-context prediction accuracy is the highest when training and testing on the same context, as expected, but not when all contexts are mixed. A similar observation can be made on the cross-context prediction accuracy for AMPS without annotation (Supplementary Figure S14). Also observe that training on CG overall allows good predictions on CHG, and vice versa; CHH seems quite different, which is supported by studies that show the molecular mechanisms for CHH are distinct from those for CG and CHG [see e.g. (54)]. We also carried out cross-accession experiments for two *Arabidopsis* accessions, namely Columbia-0 and C24. Supplementary Figure S17 shows the accuracy of AMPS (with annotations) when trained on one accession and tested on another. Observe that the accuracy of AMPS is quite high. In fact, the accuracy of AMPS is higher in cross-accession experiments than cross-species experiments, as expected.

### Interpretability analysis

In the sixth and last step, we carried out an interpretability analysis of the classifier using Grad-CAM, MEME and TOMTOM. Briefly, we used Grad-CAM to identify the most important 50-mer in the input window for the correct classification of the methylation status; the 50-mers were processed by MEME to compute statistically significant motifs and then MEME motifs were matched against known motifs in the plant motif database JASPAR using TOMTOM (see the ‘Materials and Methods’ section for details).

**Figure 3.**
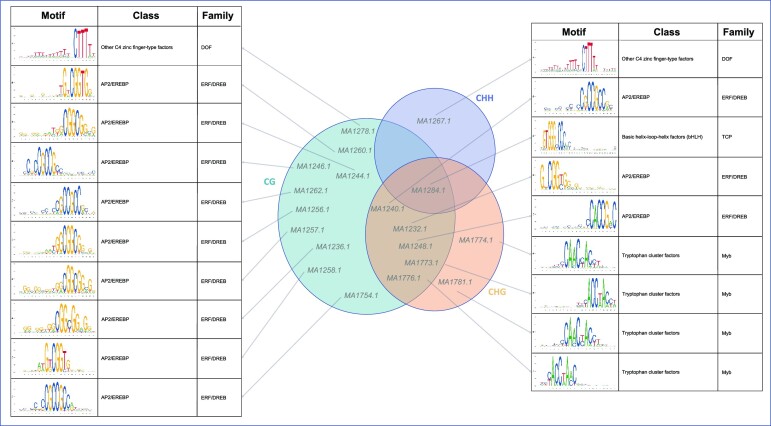
Statistically significant motifs that are critical for the accurate prediction of methylated and unmethylated cytosines in all contexts and all species.

Figure 3 lists all the statistically significant motifs found by our analysis in all species that matched JASPAR (those on the left are for the CG context and those on the right are for CHH/CHG). The Venn diagram in the middle shows the assignment of motifs to different contexts. Observe that most of the motifs are for the CG context. Also observe that almost all the motifs for the CG context are in the AP2/EREBP (ethylene-responsive element binding proteins) class, which have been shown to affect DNA methylation in plants (55,56). More specifically, Zhu *et al.* (56) showed that under drought stress, cytosine methylation is altered in the promoter region of genes containing the AP2/EREBP domain. López *et al.* (57) showed that under heat stress, 31% of the 99 transcription factor genes associated with differentially methylated regions in the strawberry genome had the AP2/EREBP domain. The MA1284.1 motif (in common to all contexts) is the structural motif for a basic helix–loop–helix, which belongs to a family of transcription factors whose binding is known to be inhibited by DNA methylation [see e.g. (58)]. One of the listed motifs in Figure 3 is the binding site for zinc finger-type factors that are known to be readers of methylated DNA (59). We could not find any relation between the tryptophan cluster factors and DNA methylation in the literature.

### Prediction based on neighboring cytosines

**Figure 4.**
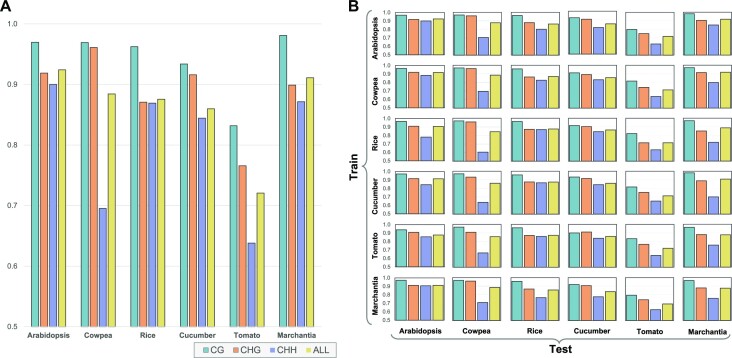
(**A**) Methylation prediction accuracy from the methylation levels of the neighboring cytosines. (**B**) Cross-species methylation prediction accuracy from the methylation levels of the neighboring cytosines.

In this section, we studied the problem of predicting cytosine methylation from the methylation levels of the neighboring cytosines, which is common in the literature for data imputation. In this case, the classifier took in input a vector of methylation levels (half upstream and half downstream, under the condition that cytosines had to have a sufficient read coverage to be included) and predicted the binary methylation status of the center cytosine. In all experiments, we used 20 methylation levels (10 downstream and 10 upstream). The data set size was 50 000 methylation vectors uniformly sampled from the genome, in which half of them were centered at a methylated cytosine while the other half were centered at an unmethylated cytosine. Eighty percent of the data set was used for training, 10% was used for validation and 10% was used for testing. Our classifier was a fully connected neural network with four hidden layers (more details are provided in the ‘Materials and Methods’ section). As we did earlier, we carried out methylation prediction for each species and for each context individually, but also for all contexts combined. Figure 4A shows that the prediction accuracy is again context- and species-specific. More specifically, observe that (i) cytosine methylation in the CG context is the easiest to predict, while methylation in the CHH context is the hardest (somewhat the opposite of what we observed for sequence-based prediction, as shown in Figure 1), (ii) combining the contexts degrades the prediction performance compared to context-specific classifiers and (iii) methylation prediction in tomato appears to be harder than other species. Also observe that the accuracy appears to be correlated with the average cytosine coverage (Supplementary Table S3). For instance, the worst overall accuracy is for tomato, which has the lowest average cytosine coverage. The best overall accuracy is for *Arabidopsis*, which has the highest average cytosine coverage.

We also carried out cross-species and cross-context predictions using neighboring cytosines. Figure 4B shows the prediction accuracy when training on a species and testing on another. Observe that the accuracy does not change significantly as one moves down the rows of the matrix. This indicates that the prediction accuracy is somewhat independent of the trained species, which is again different from what we observed for sequence-based prediction (as shown in Figure 2A). Supplementary Figure S15 shows the prediction accuracy when training on a context and testing on another. With some exceptions, observe again that the accuracy does not significantly change as one moves down the rows. This implies that the prediction accuracy is somewhat independent of the trained context.

Finally, we investigated the predictive performance of AMPS when providing in input (i) the sequence, (ii) the annotations and (iii) the methylation levels of neighboring cytosines. The architecture of this classifier is described in the ‘Classifiers’ section. Supplementary Figure S16 compares the prediction accuracy obtained from methylation levels of neighboring cytosines to the accuracy obtained when the sequence, annotations and methylation levels of neighboring cytosines are used. Observe that (i) in most cases, the accuracy did not significantly improve when sequence and annotations were provided, (ii) the accuracy improvement on tomato was significant and (iii) in two cases (for the ALL context), the accuracy decreased when sequence and annotations are used.

### Effect of the window size and training set size on the prediction accuracy

Two critical parameters for the prediction accuracy from the DNA sequence are (i) the size of the training set and (ii) the size of the input sequence (or *window size*). Here, we carried out extensive tests to determine the optimal values for these two parameters using AMPS (with annotations) as a classifier.

As expected, the size of the training set directly affects the performance of the classifier. We recorded the accuracy of AMPS (with annotations) on all species and all contexts using a data set with 40k, 80k, 120k, 200k, 400k, 600k, 800k and 1M sequences. Eighty percent of the data was used for training, 10% was used for validation and 10% was used for testing. For some (organism, context) pairs, the number of cytosines that had sufficient read coverage to be called methylated (or not) was insufficient to satisfy the data set needs. In those cases, the larger data sets are missing from the analysis and the figures. To investigate the extent of variations induced by the random sampling of the training set, we carried out 10 replicates on all contexts in *Arabidopsis* and recorded average and standard deviation of AMPS accuracy (see Supplementary Figure S7). Observe that the standard deviation is very low, which allowed us to avoid replicates (and thus save on compute time) for all other experiments in this manuscript.

Supplementary Figure S5 illustrates AMPS’ accuracy as a function of the data set size for all plant species. Observe that for data set with 400 000 sequences or more, the accuracy is high and relatively stable in all plant species. Based on this observation, we used data sets composed of 500 000 sequences, if there were sufficient cytosines available. If there were not, we used all the available cytosines.

Supplementary Figure S6 shows AMPS’ accuracy as a function of the window size (100, 200, 400, 800, 1600, 3200 and 6400 bp) for all plant species. Observe that context-specific predictions are differently affected by the window size. For CG and CHG, the prediction accuracy increases up to a window size of 3200 bp. However, for CHH the accuracy does not change or degrade by increasing the window size. We do not have an explanation for this phenomenon.

## DISCUSSION

In this study, we investigated the problem of predicting cytosine methylation in plants from either the DNA sequence or the neighboring cytosines. To the best of our knowledge, this is the first time that independent predictions for different contexts and plant species have been carried out and compared. We can summarize our findings in three major categories.

Our first finding is that the cytosine methylation prediction from the sequence is more accurate when a context-specific species-specific classifier is used. Combining the contexts during training, which is what most studies in the literature have done so far (although some focus only on CG), degrades the classifier’s performance. Our study suggests that context-specific species-specific predictive models are necessary for obtaining the best overall predictive performance for cytosine methylation from the primary sequence in plants, and possibly in other organisms. This is true whether annotations are used or not.

The second finding is that the predictive accuracy of cytosine methylation from the methylation levels of neighboring cytosines is higher than the predictive accuracy obtained from the sequence only (with or without annotation). This is consistent with results reported in the literature for other organisms (mostly vertebrates). However, to the best of our knowledge, no study has compared predictions from neighboring cytosines across multiple organisms or across contexts. In fact, cross-accession, cross-species and cross-context prediction appears sufficiently accurate, which opens the possibility of methylation imputation across species or accessions, especially when annotations are available. While imputation for a small fraction of genome-wide cytosines is feasible, we would be very cautious using methylation predictions for an entire new genome based on training the classifier on a related species. Interestingly, while the easiest context to predict from the sequence is CHG, the easiest context to predict from neighboring methylation levels is CG.

The final finding of our study is that using annotation data (gene and repeat location) dramatically improves the predictive accuracy of cytosine methylation from the sequence, not only for our classifier but also for all the classifiers that we could instrument with this additional layer of information. While this finding is not completely surprising, the extent of the improvement is striking.

## DATA AVAILABILITY

BS-Seq data supporting the conclusions of this article are available in the NCBI SRA repository with accession numbers SRR3171614, SRR618545, SRR618546, SRR618547, SRR503393 and SRR5430777 for *Arabidopsis*, rice, cucumber and tomato, respectively. BS-Seq data for marchantia are available in the NCBI SRA repository with accession numbers SRR5314027, SRR5314028, SRR5314029, SRR5314030, SRR5314031 and SRR5314032. Cowpea BS-Seq data were deposited in the European Nucleotide Archive with accession number PRJEB52355. Code and scripts are available in the public GitHub repository: https://github.com/ucrbioinfo/AMPS (permanent doi: 10.5281/zenodo.7894703).

## Supplementary Material

lqad045_Supplemental_File
