# Supplementary material for: On the prediction of non-CG DNA methylation using machine learning
Source: NAR Genom Bioinform. 2023 May 17;5(2):lqad045. doi: 10.1093/nargab/lqad045 (PMC10189801; doi:10.1093/nargab/lqad045)
Supplement: lqad045_Supplemental_File [file lqad045_supplemental_file.pdf]

On the Prediction of non-CG DNA Methylation using Machine Learning

Supplementary material

Saleh Sereshki, Nathan Lee, Michalis Omirou, Dionysia Fasoula and Stefano Lonardi

Supplementary Tables

species	source	assembly version	size (Mb)
Arabidopsis	The Arabidopsis Information Resource	TAIR10.1	119.6
Cowpea	JGI Phytozome	IT97K-499-35 v.1	519.4
Rice	International Rice Genome Sequencing Project	IRGSP-1.0	373.2
Cucumber	The Cucumber Genome Initiative	GCF_000004075.3_9930_V3	226.6
Tomato	Solanaceae Genomics Project	GCF_000188115.4_SL3.0	828.3
Marchantia	NCBI genome database	GCA_003032435.1	225.7

Supplementary Table 1: Source, version and genome size of the genome assemblies used in this study

species	SRA source	number of reads	% of reads mapped	read length (after trimming)	read type
Arabidopsis	SRR3171614	209,561,030	54.40%	50	single end
Cowpea	PRJEB52355	141,137,614	39.30%	150	paired end
Rice	SRR618545-7	622,913,368	47.30%	50 (37)	single end
Cucumber	SRR5430777	59,999,999	50.10%	90	paired end
Tomato	SRR503393	84,127,751	77.70%	101 (91)	paired end
Marchantia	SRR5314027-32	163,128,361	29.21%	126 (119)	paired end

Supplementary Table 2: Summary of the BS-Seq data sets used in this study: SRA/EMBL source, number of reads, read length (before and after trimming), read type and % of reads uniquely mapped

species	genome size (Mb)	average read coverage	average cytosine coverage
Arabidopsis	119.6	48x	21x
Cowpea	519.4	32x	12x
Rice	373.2	29x	12x
Cucumber	226.6	24x	9x
Tomato	828.3	16x	5x
Marchantia	225.7	50x	17x

Supplementary Table 3: Genome sizes, average genome coverage from Bismark mapped reads, average cytosine coverage from Bismark mapped reads

		methylated cytosine (%)			
species	Cs with sufficient coverage (%)	CG	CHG	CHH	ALL
Arabidopsis	62.29%	27.54%	7.72%	0.68%	6.00%
Cowpea	44.97%	60.40%	47.54%	4.41%	16.30%
Rice	37.87%	54.15%	26.87%	2.66%	18.21%
Cucumber	22.63%	56.58%	24.93%	5.22%	15.05%
Tomato	9.13%	89.56%	62.88%	2.73%	19.01%
Marchantia	45.18%	33.92%	10.87%	0.17%	7.38%

Supplementary Table 4: Summary of cytosine methylation statistics for the species in this study; the second column shows the percentage of cytosines that have a coverage of more than ten reads after mapping BS-Seq reads with Bismark; the rest of the columns show the percentage of methylated cytosines in each individual context (only for the cytosines that had sufficient coverage)

species	number of annotated elements
Arabidopsis	genes (38,310), exons (324,656), CDS (286,173), repeats (73,682)
Cowpea	genes (31,947), CDS (325,882), repeats (646,218)
Rice	mRNA (44,784), exons (198,572), repeats (420,201)
Cucumber	gene (23,636), exons (254,378), CDS (201,484), repeats (268,695)
Tomato	genes (30,017), exons (310,488), CDS (235,394), repeats (417,920)
Marchantia	genes (19,287), exons (150,201), CDS (137,019), repeats (166,589)

Supplementary Table 5: Number of available annotations in each species

	methylated				unmethylated			
species	CG (%)	CHG (%)	CHH (%)	ALL (%)	CG (%)	CHG (%)	CHH (%)	ALL (%)
Arabidopsis	72.61	20.57	6.82	100	11.85	16.26	71.88	100
Cowpea	40.79	40.23	18.97	100	5.23	8.76	86.01	100
Rice	63.91	27.95	8.13	100	11.88	17.05	71.07	100
Cucumber	52.59	24.28	23.13	100	6.93	12.82	80.25	100
Tomato	51.15	39.11	9.74	100	1.40	5.36	93.25	100
Marchantia	71.87	23.28	4.85	100	14.10	16.76	69.14	100

Supplementary Table 6: Distribution of methylated and unmethylated cytosines for each individual context

Supplementary Figures

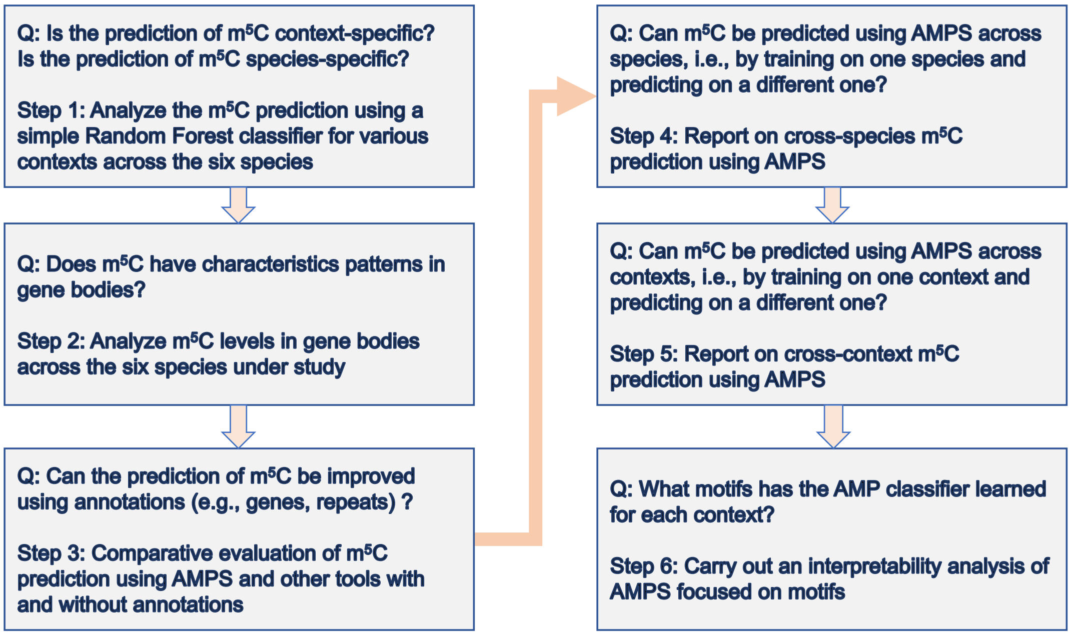

Supplementary Figure 1: The logical organization of the questions and analyses carried out in our study.

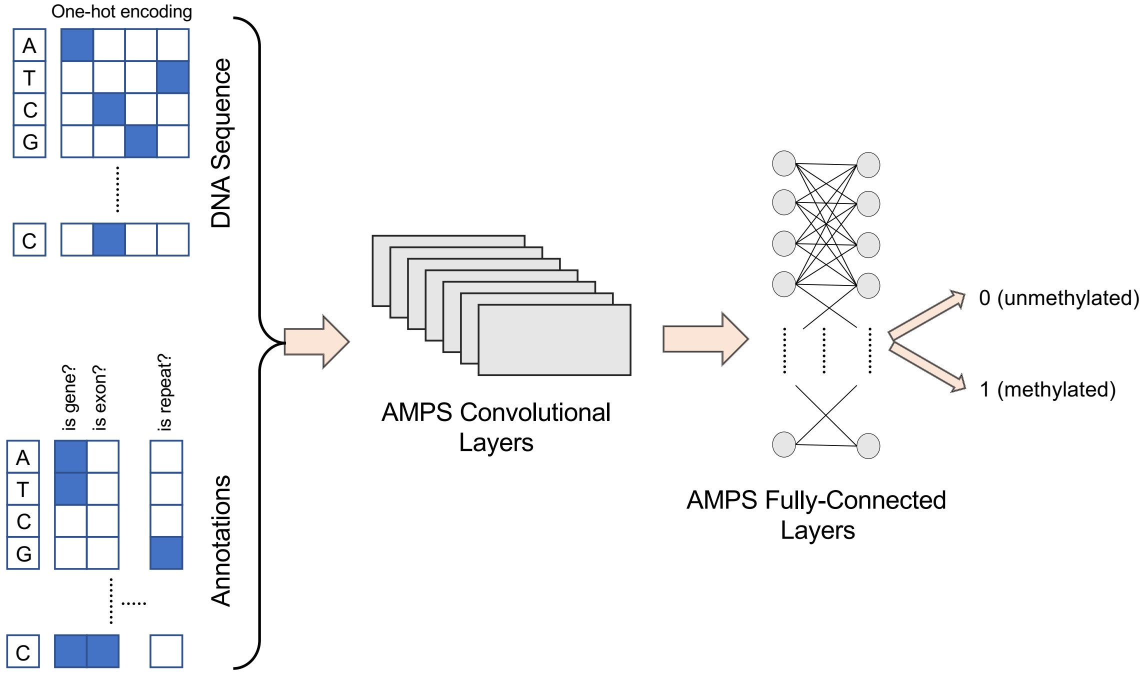

Supplementary Figure 2: The deep learning architecture of AMPS

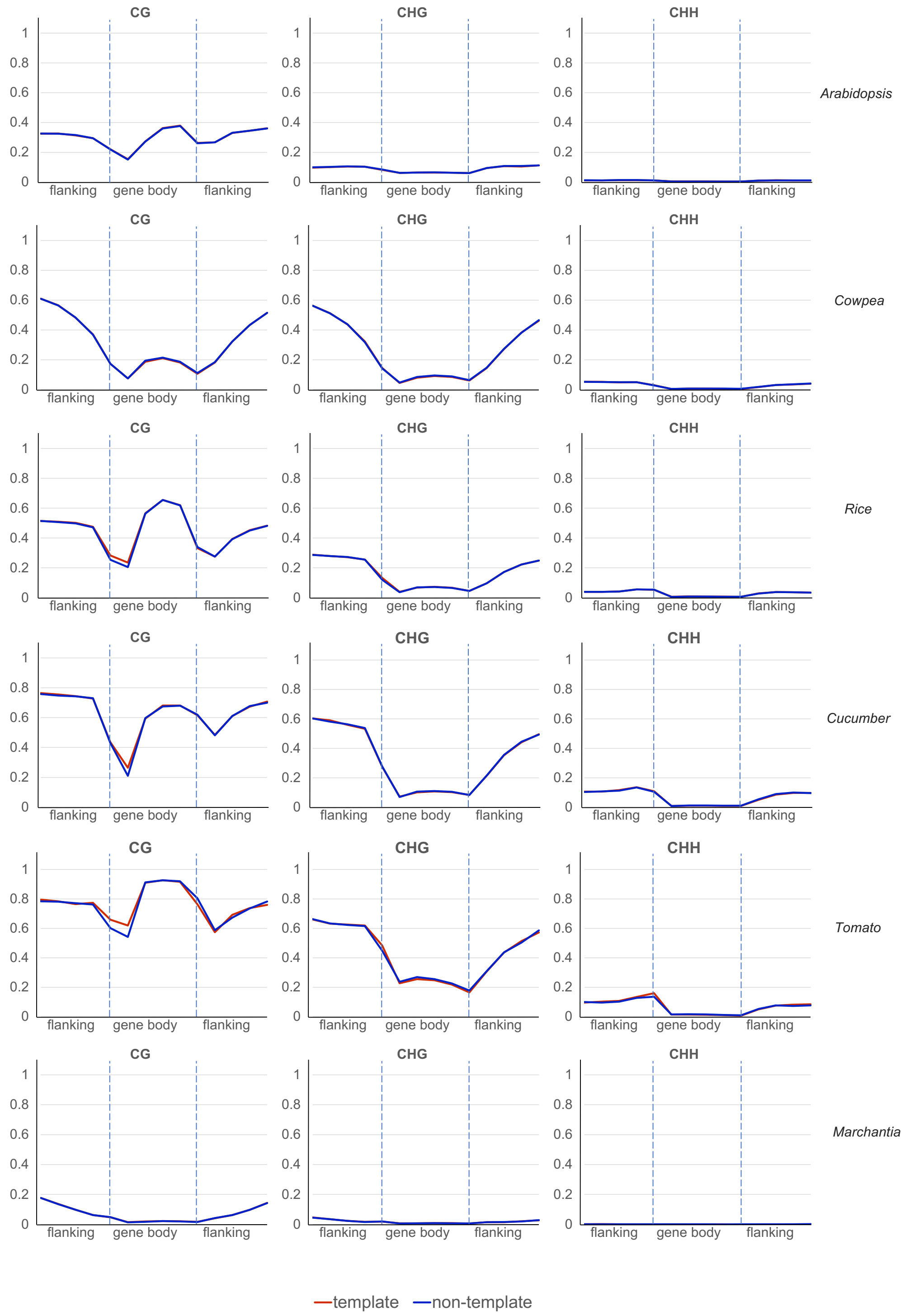

Supplementary Figure 3: Context-specific, species-specific gene body methylation levels in gene bodies and 2000 bp flanking regions (upstream and downstream) for template and non-template strands, when averaged over all genes

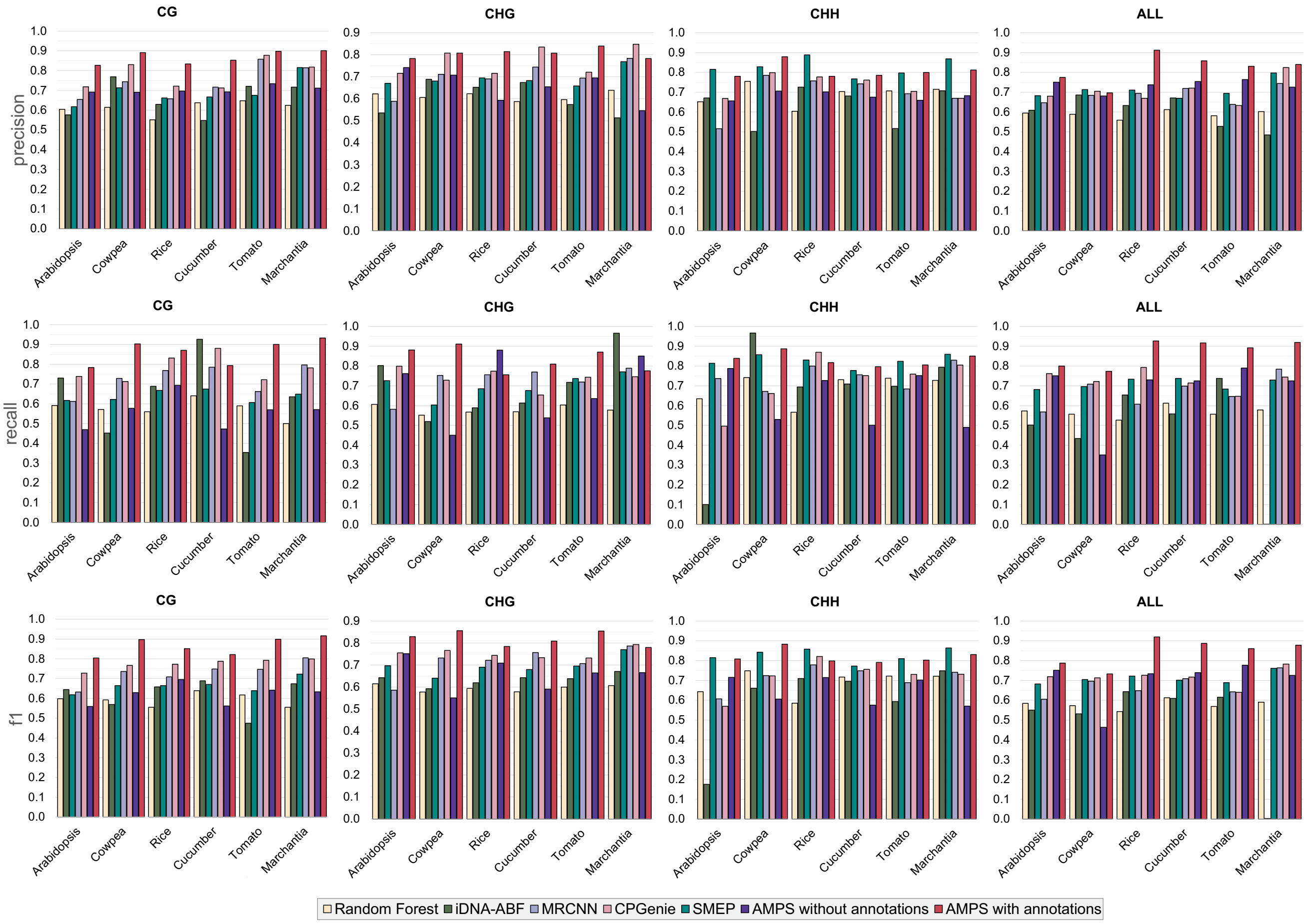

Supplementary Figure 4: Context-specific species-specific prediction F1-score, precision, and recall for Random Forest, iDNA-ABF, MRCNN, CPGenie, SMEP, AMPS without annotation, and AMPS with annotation.

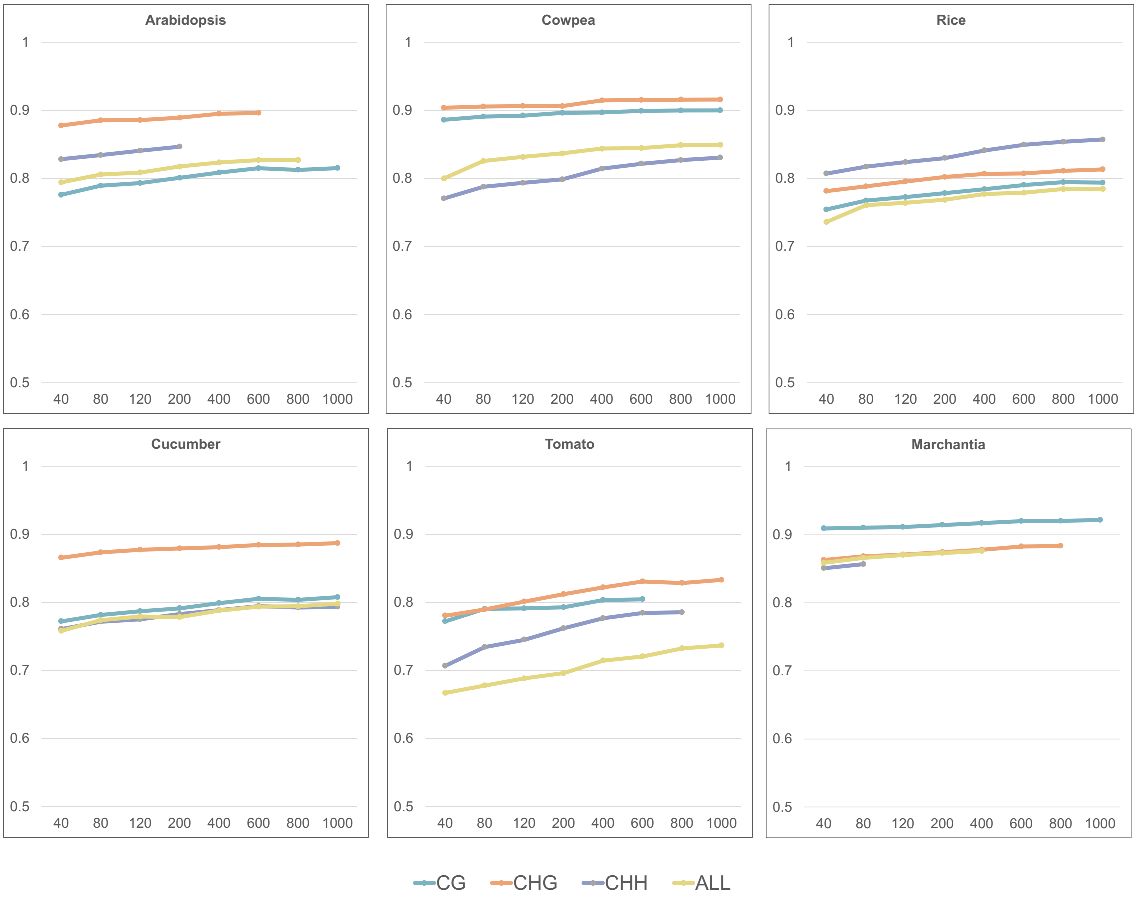

Supplementary Figure 5: Prediction accuracy as a function of the training set size for AMPS with annotations (the x-axis represents thousands of samples)

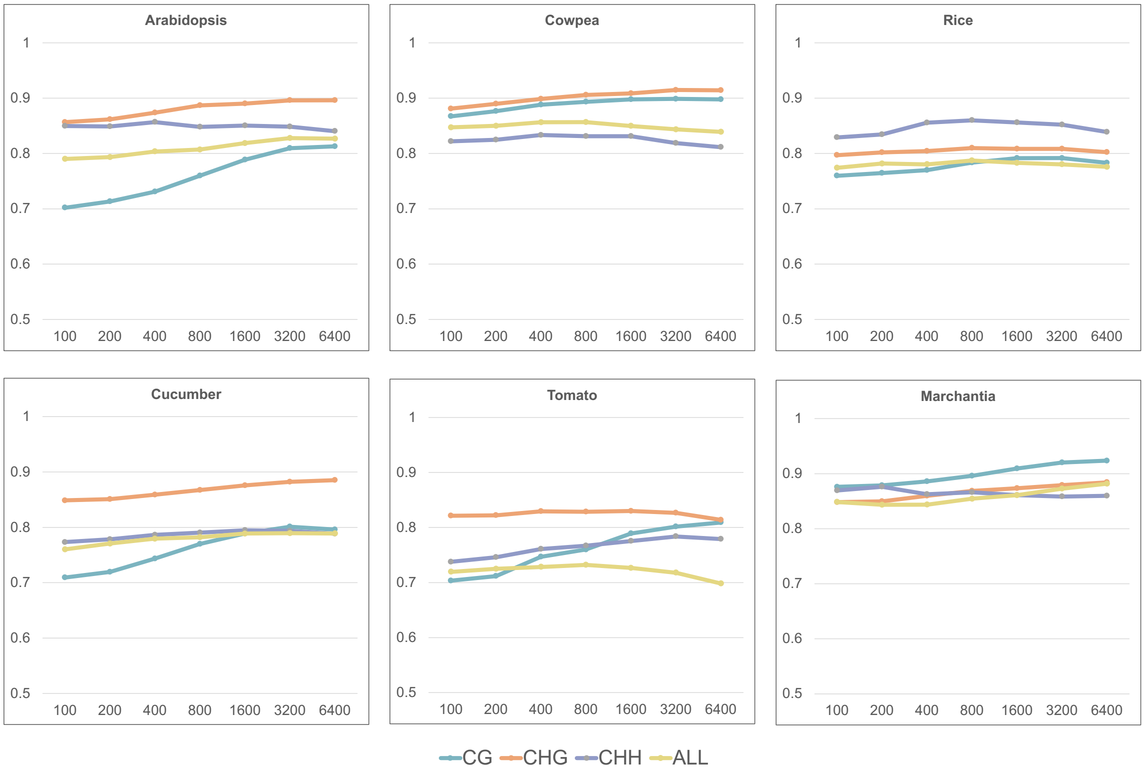

Supplementary Figure 6: Prediction accuracy as a function of the window size for AMPS with annotations (the x-axis represents the window size in base pairs)

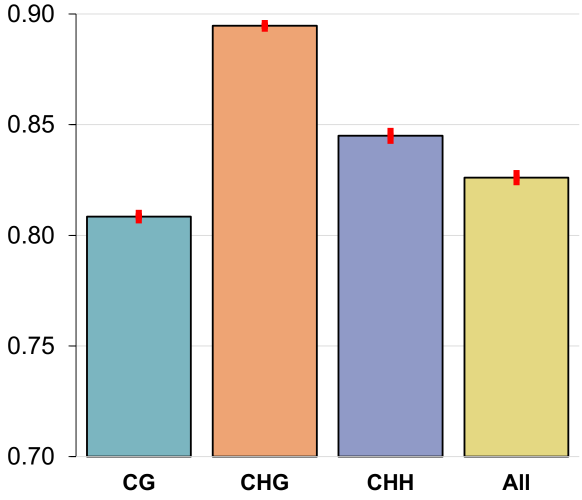

Supplementary Figure 7: Average prediction accuracy for each contexts on Arabidopsis; the error bars in red shows the standard deviation of the prediction accuracy over ten independent random samples; the standard deviations of the accuracy are 0.00173 (CG), 0.00127 (CHG), 0.00223 (CHH), and 0.00205 (ALL)

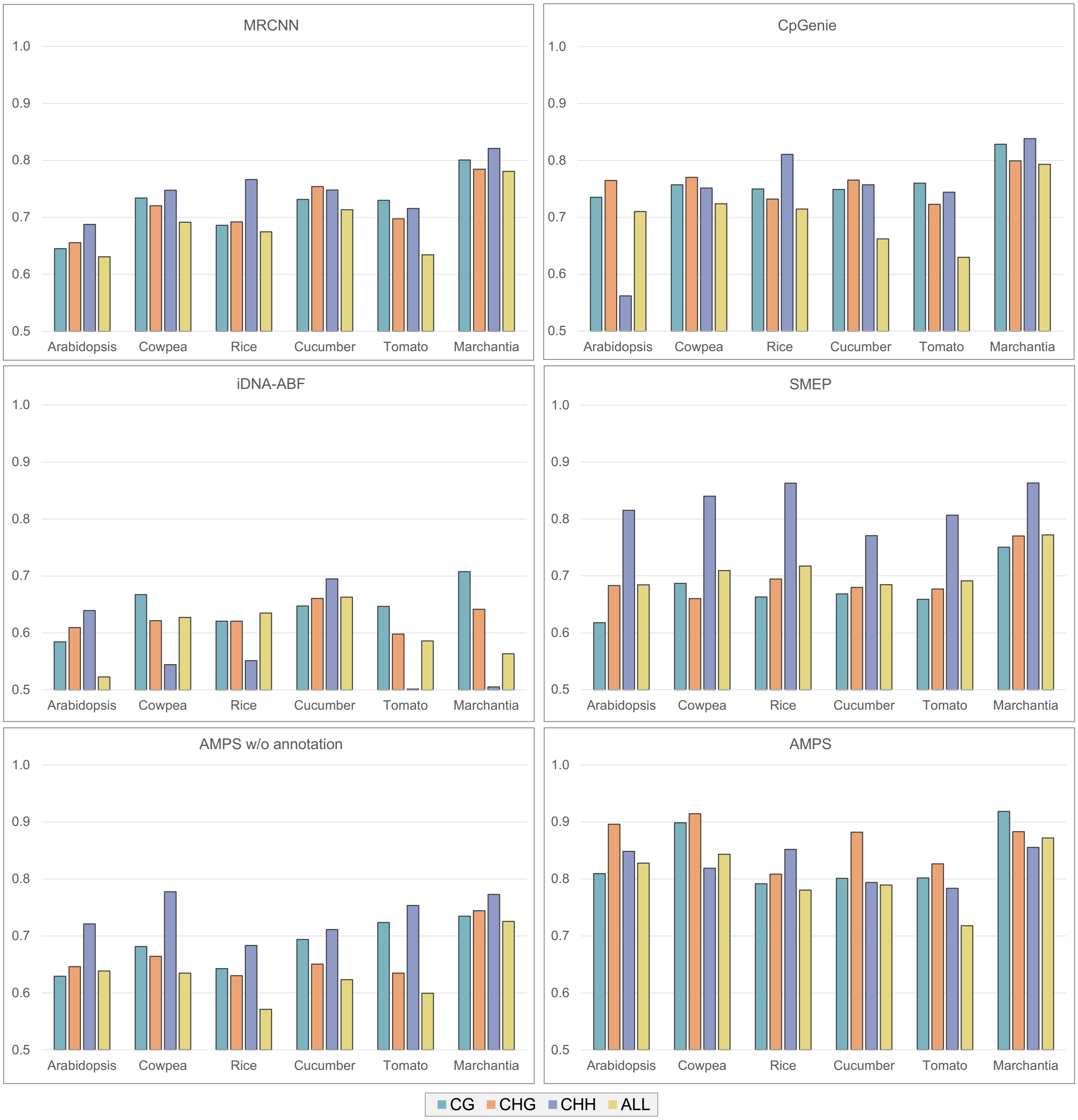

Supplementary Figure 8: Context-specific species-specific accuracy for methylation prediction from DNA sequence for MR-CNN, CPGenie, AMPS without annotations and AMPS with annotations

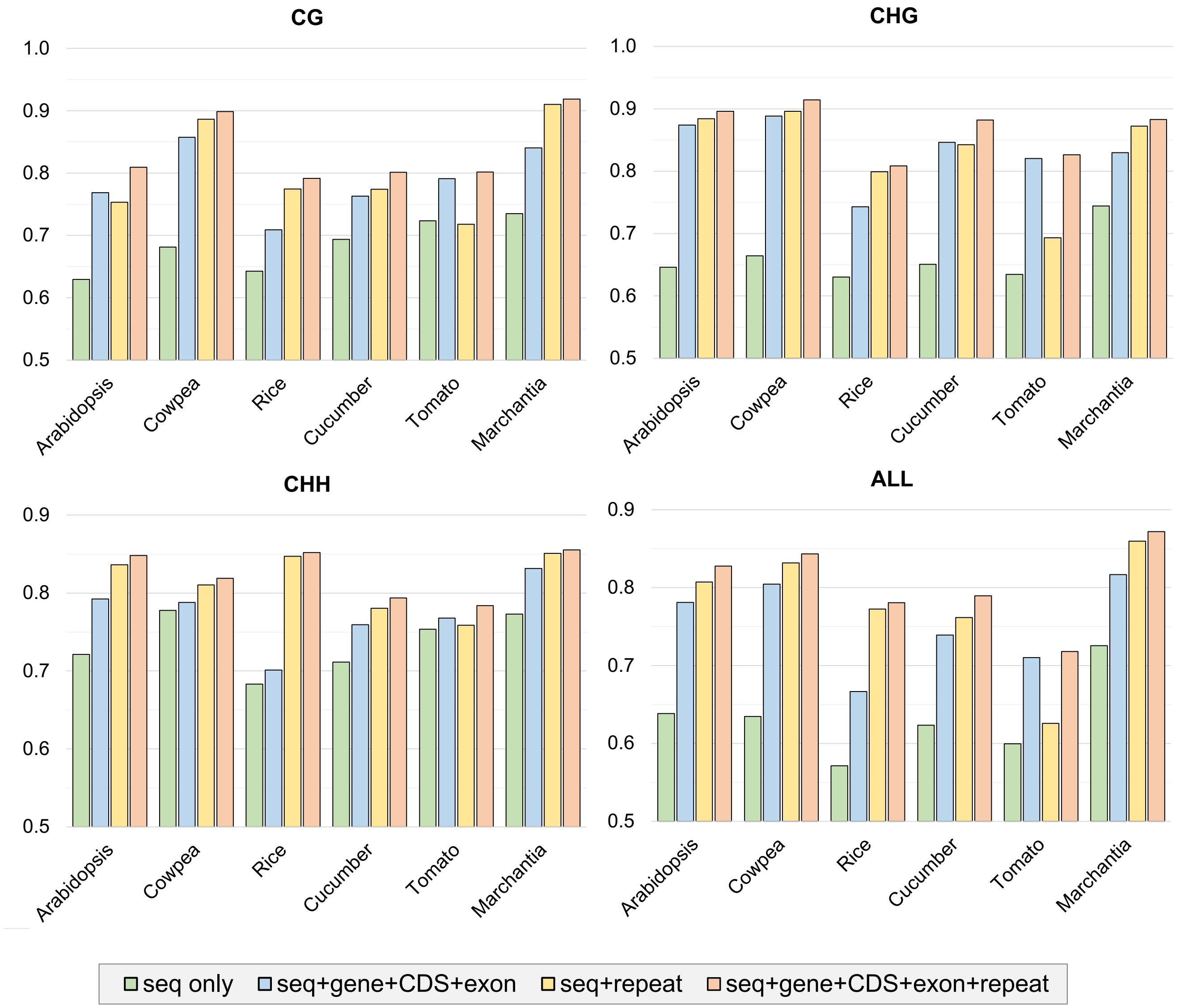

Supplementary Figure 9: Context-specific species-specific accuracy of AMPS based on the sequence only; sequence and repeat annotations; sequence and gene annotations; sequence, gene and repeat annotations

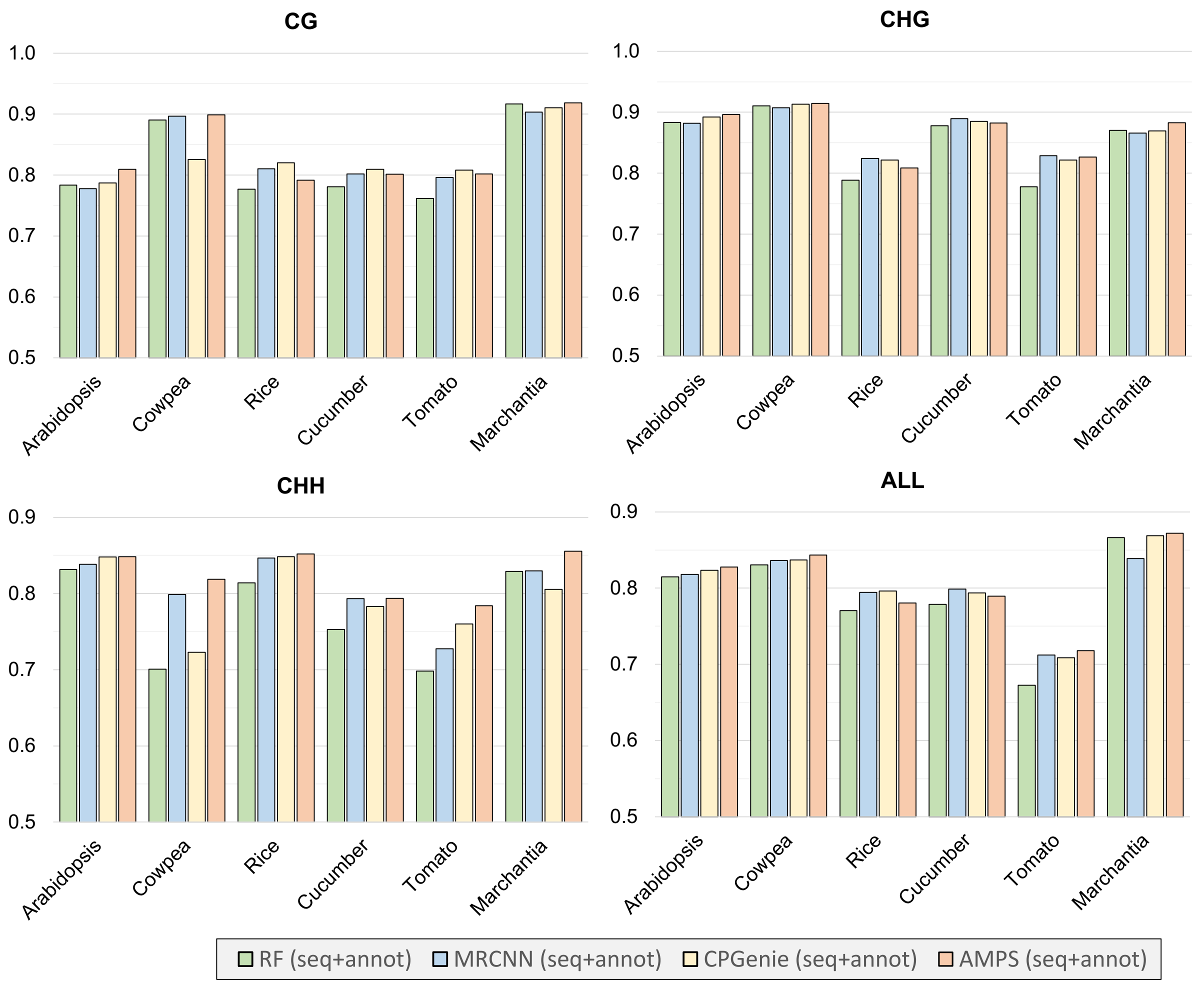

Supplementary Figure 10: Methylation prediction accuracy of Random Forest (RF), MRCNN, CPGenie and AMPS when genomic annotations are used in input, in addition to the sequence

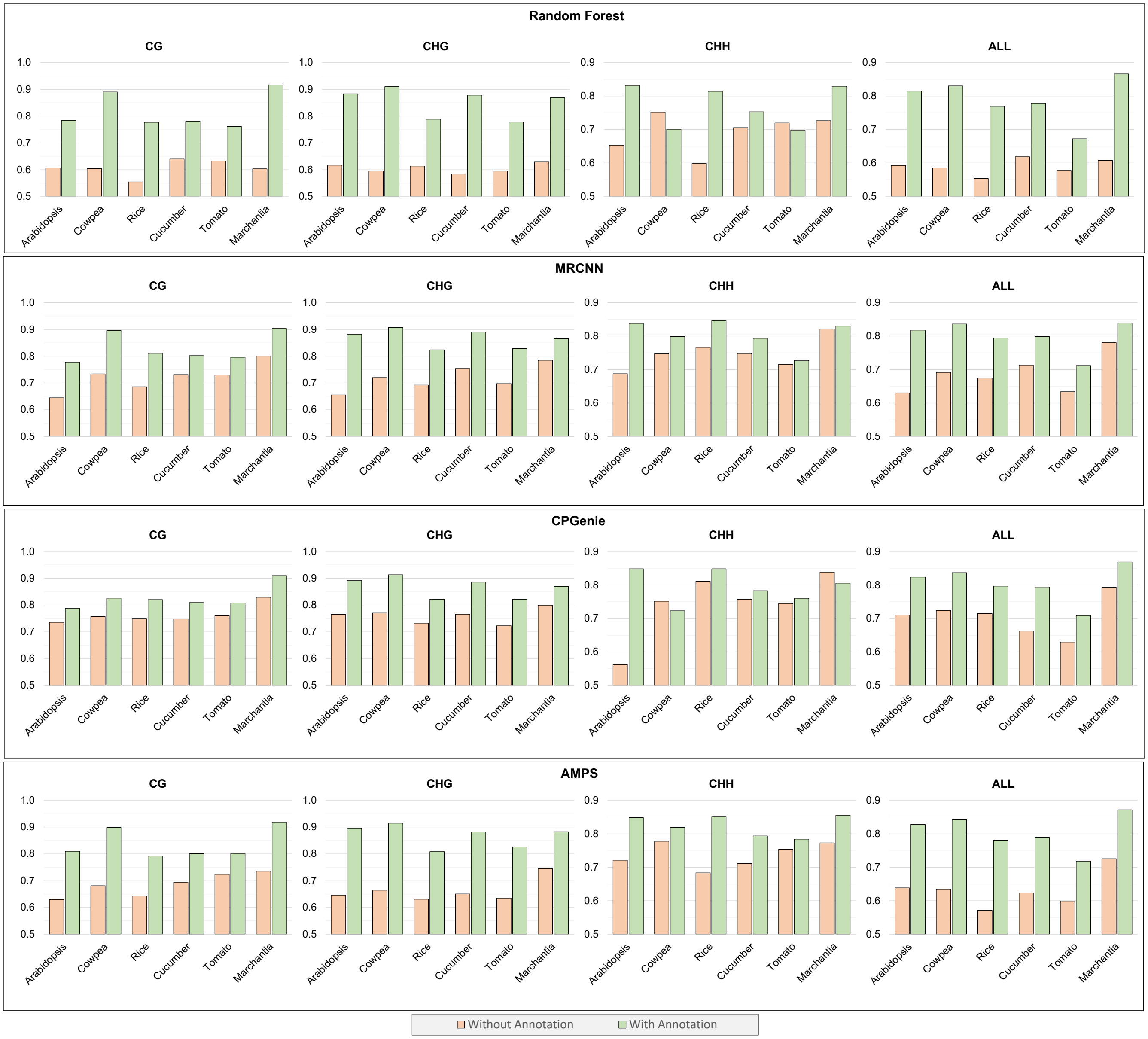

Supplementary Figure 11: Comparing the methylation prediction accuracy of Random Forest, MRCNN, CPGenie and AMPS with and without annotations

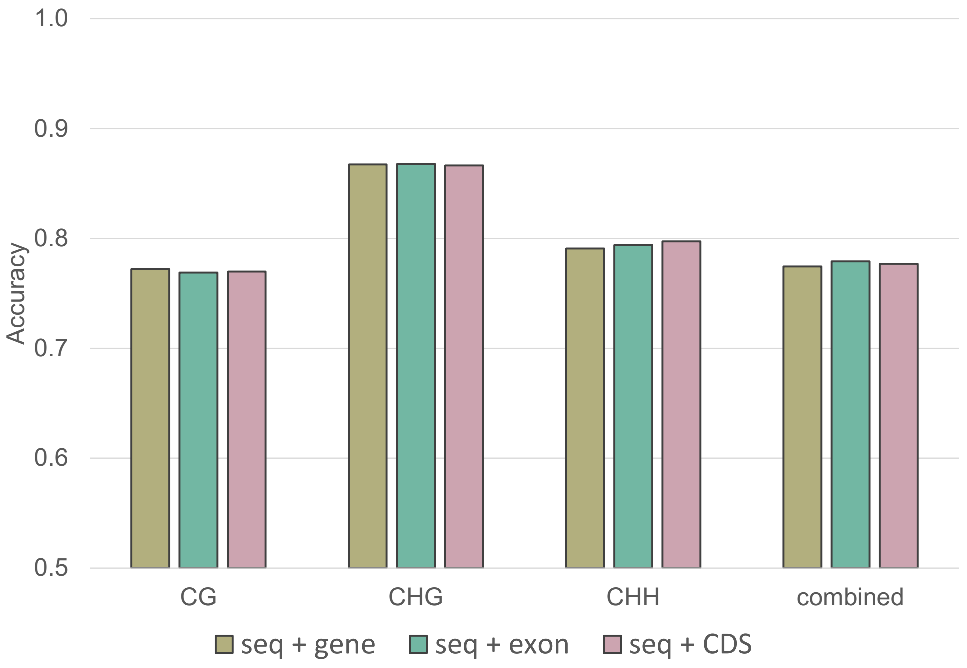

Supplementary Figure 12: Comparing the accuracy of AMPS on the Arabidopsis data set when one or more of the functional elements (annotations) are used, for different contexts

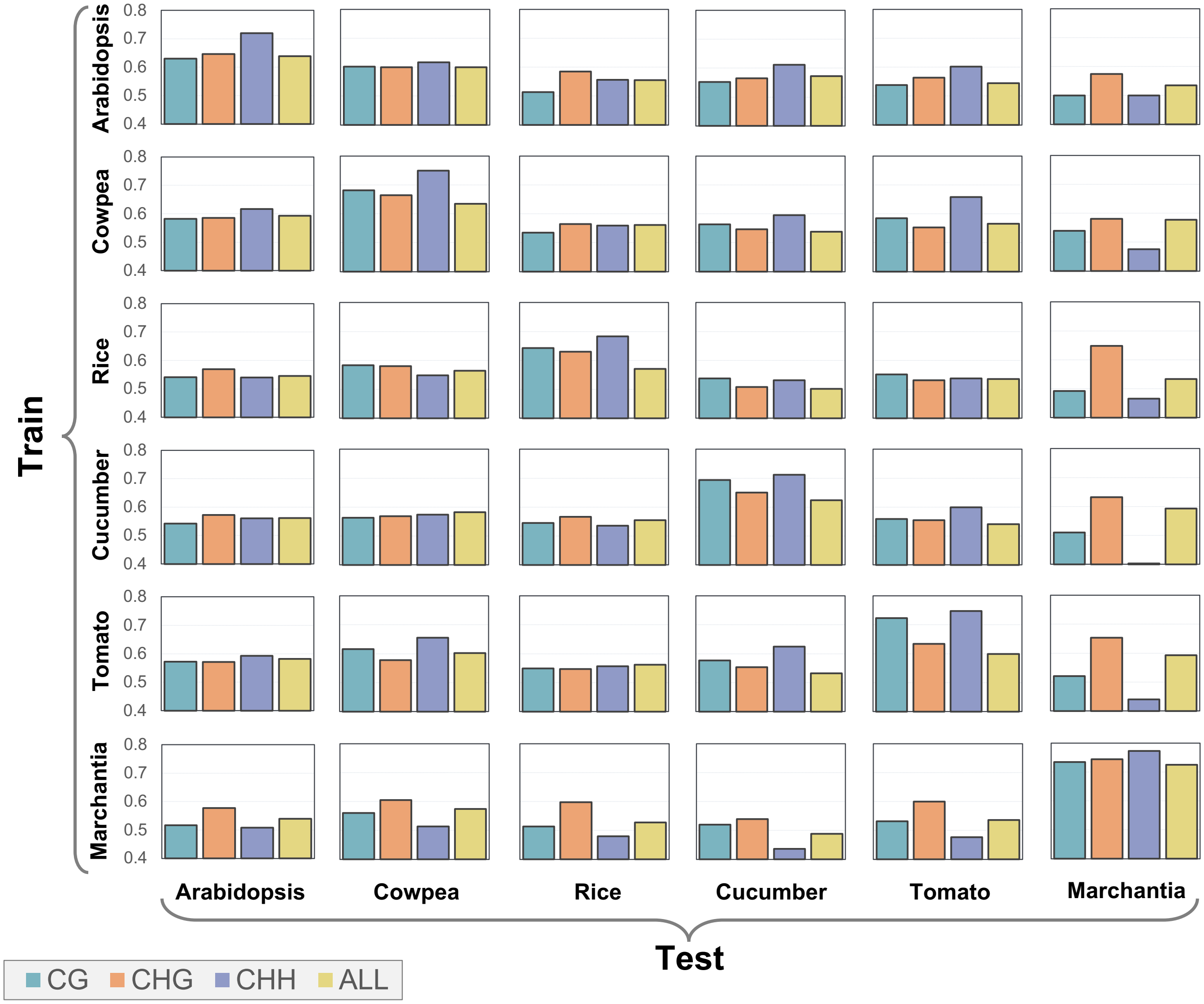

Supplementary Figure 13: Cross-species methylation prediction for AMPS without annotation

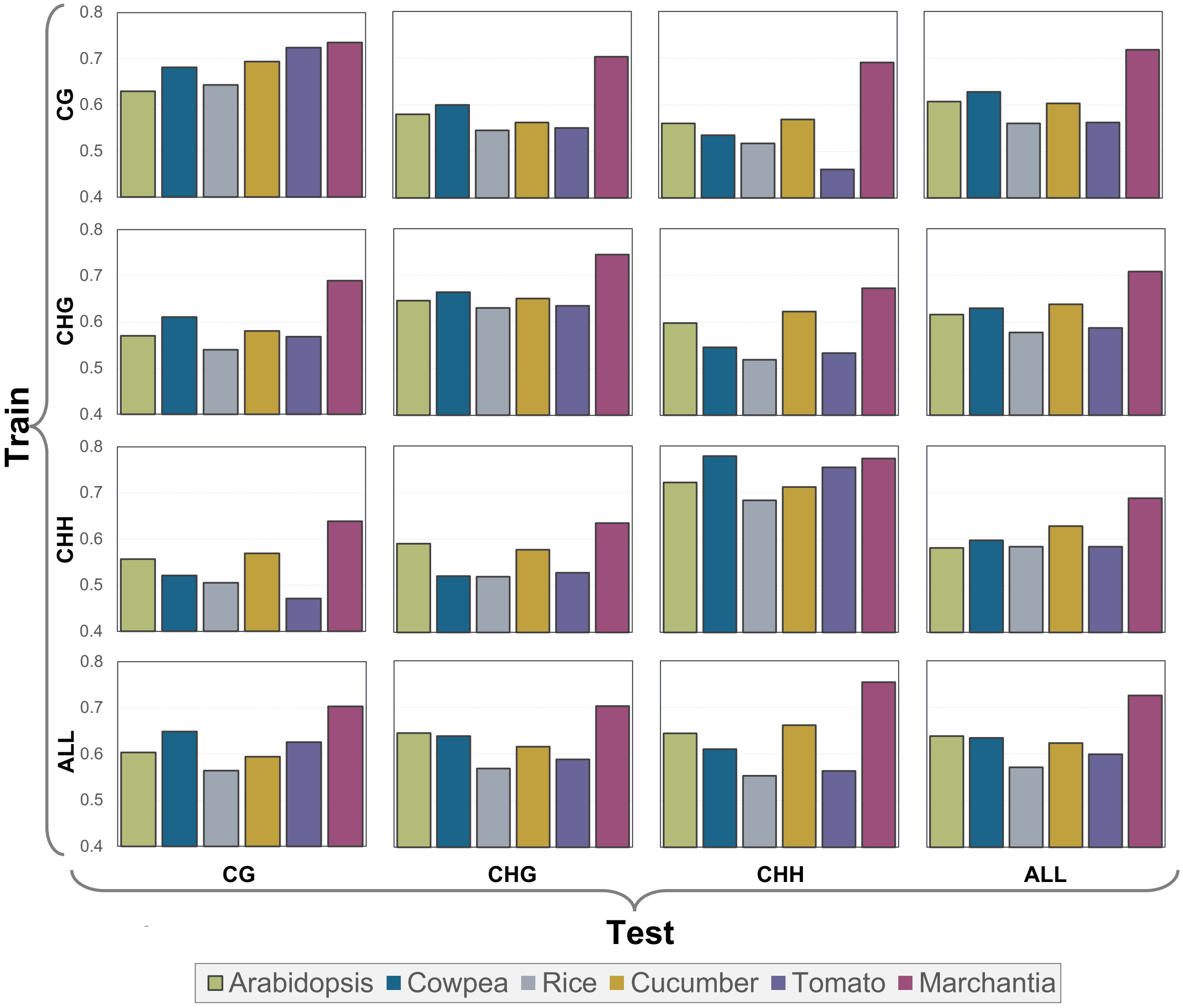

Supplementary Figure 14: Cross-context methylation prediction for AMPS without annotation

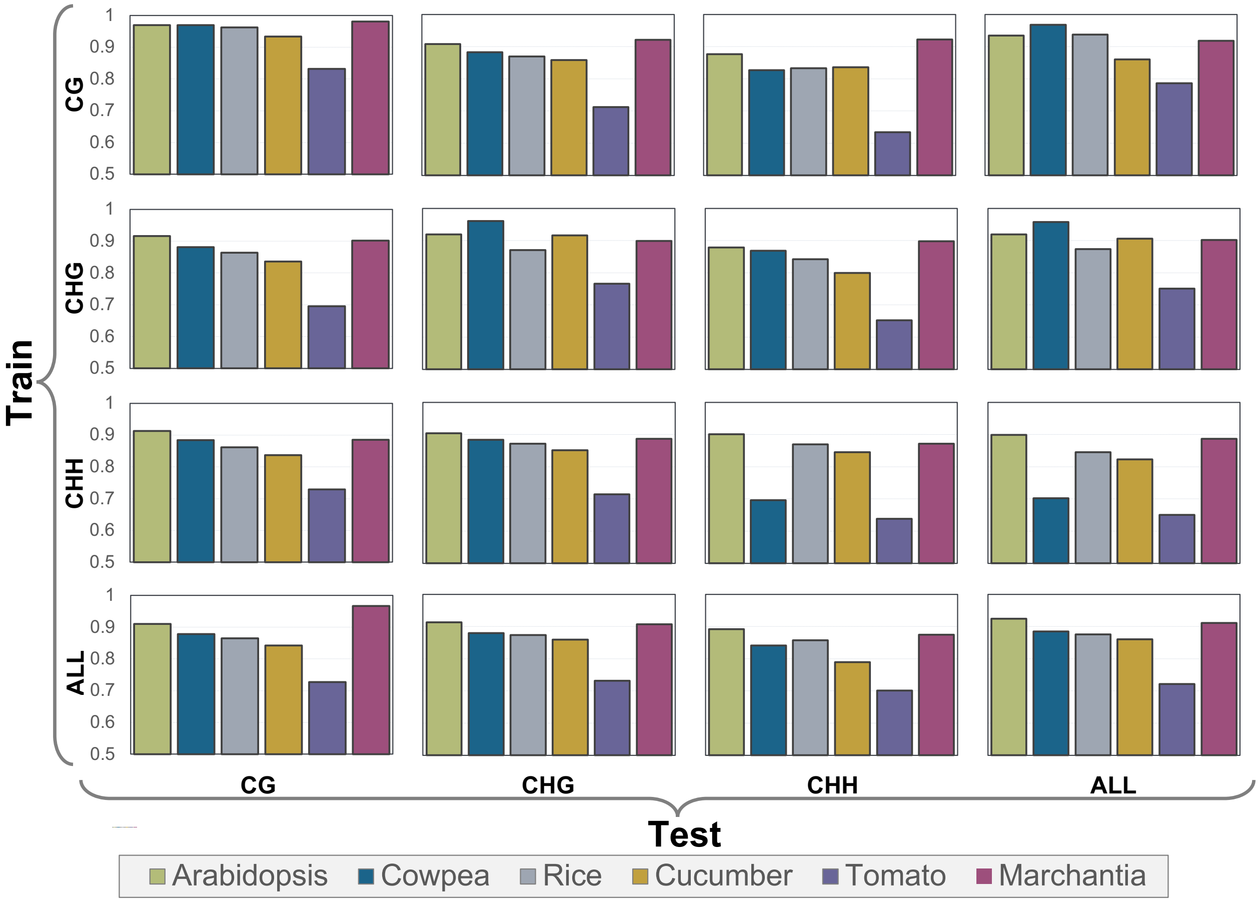

Supplementary Figure 15: Cross-context methylation prediction from neighboring cytosines

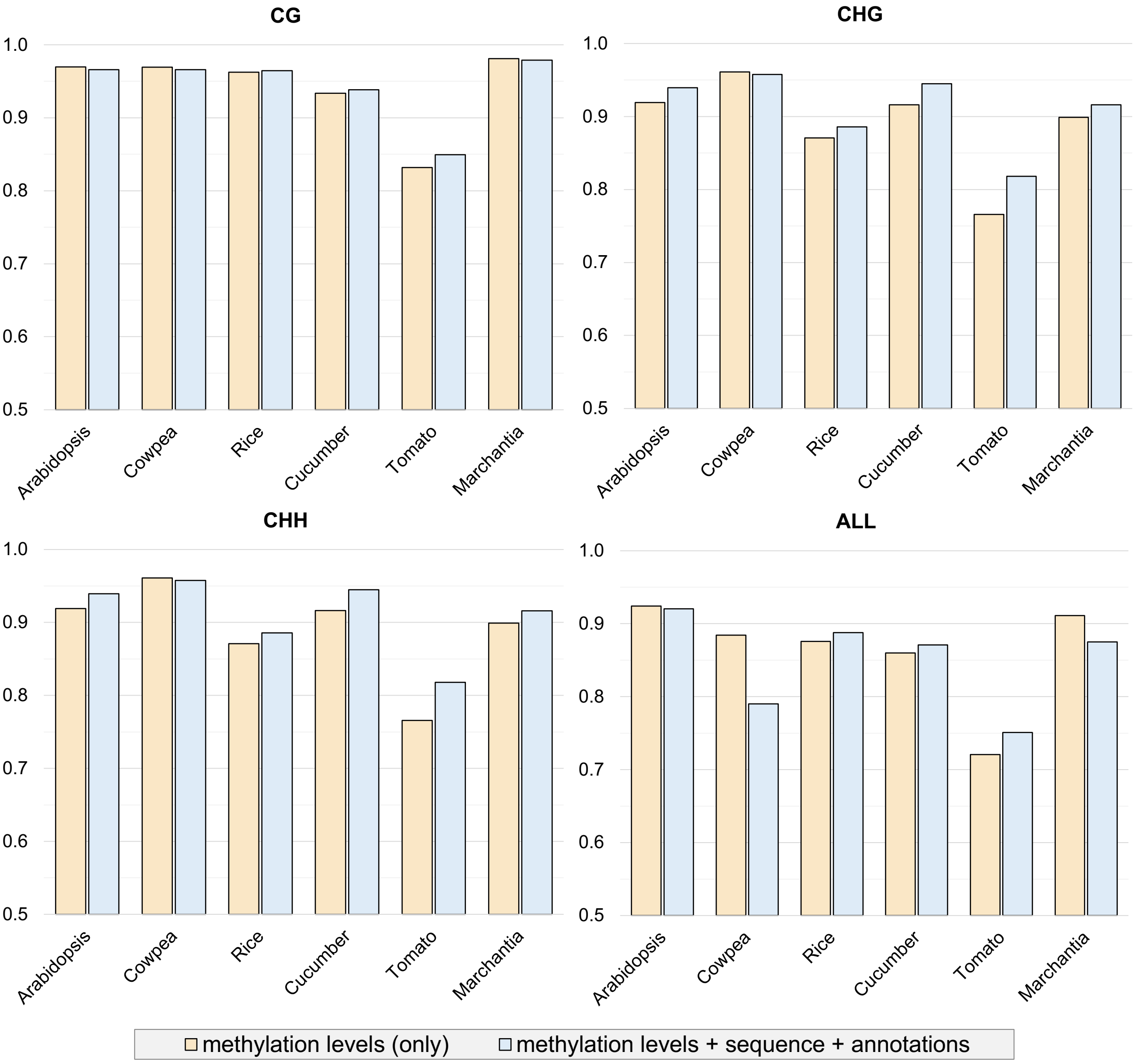

Supplementary Figure 16: Comparing the prediction accuracy of the classifier that only uses the methylation levels of neighboring cytosine to the classifiers that uses levels, sequence, and annotations

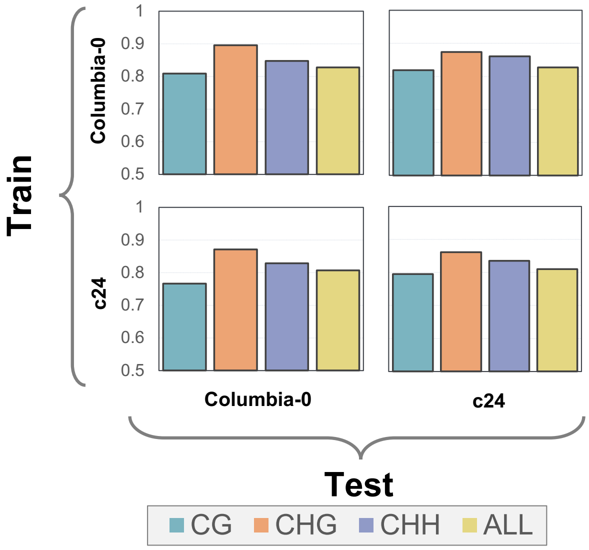

Supplementary Figure 17: Cross-accession context-specific accuracy of AMPS (with annotations) on two accessions of Arabidopsis, namely Columbia-0 and C24

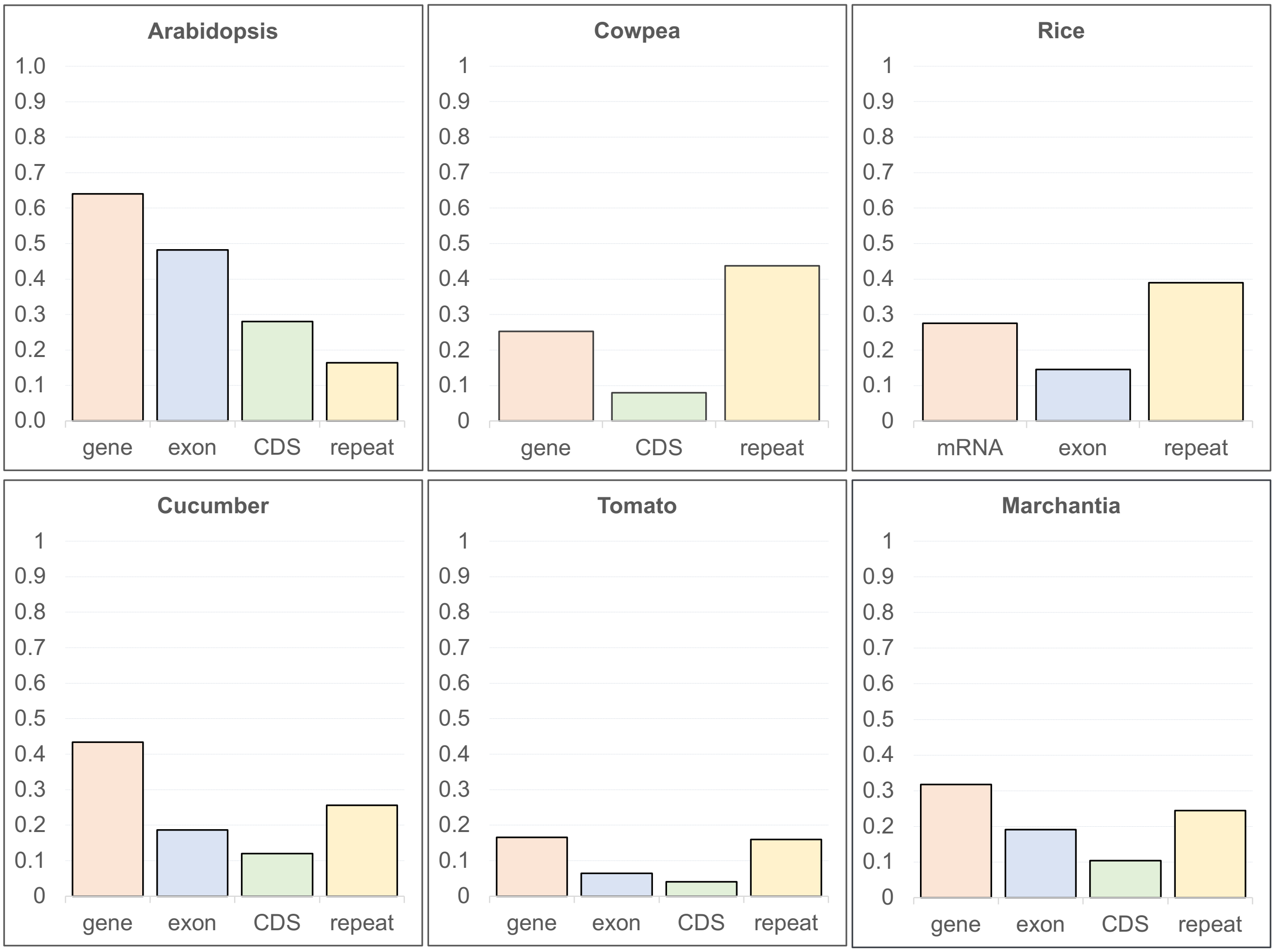

Supplementary Figure 18: Fraction of each genome covered by a functional annotation (see Supplemental Table 5 for the list) and repeats

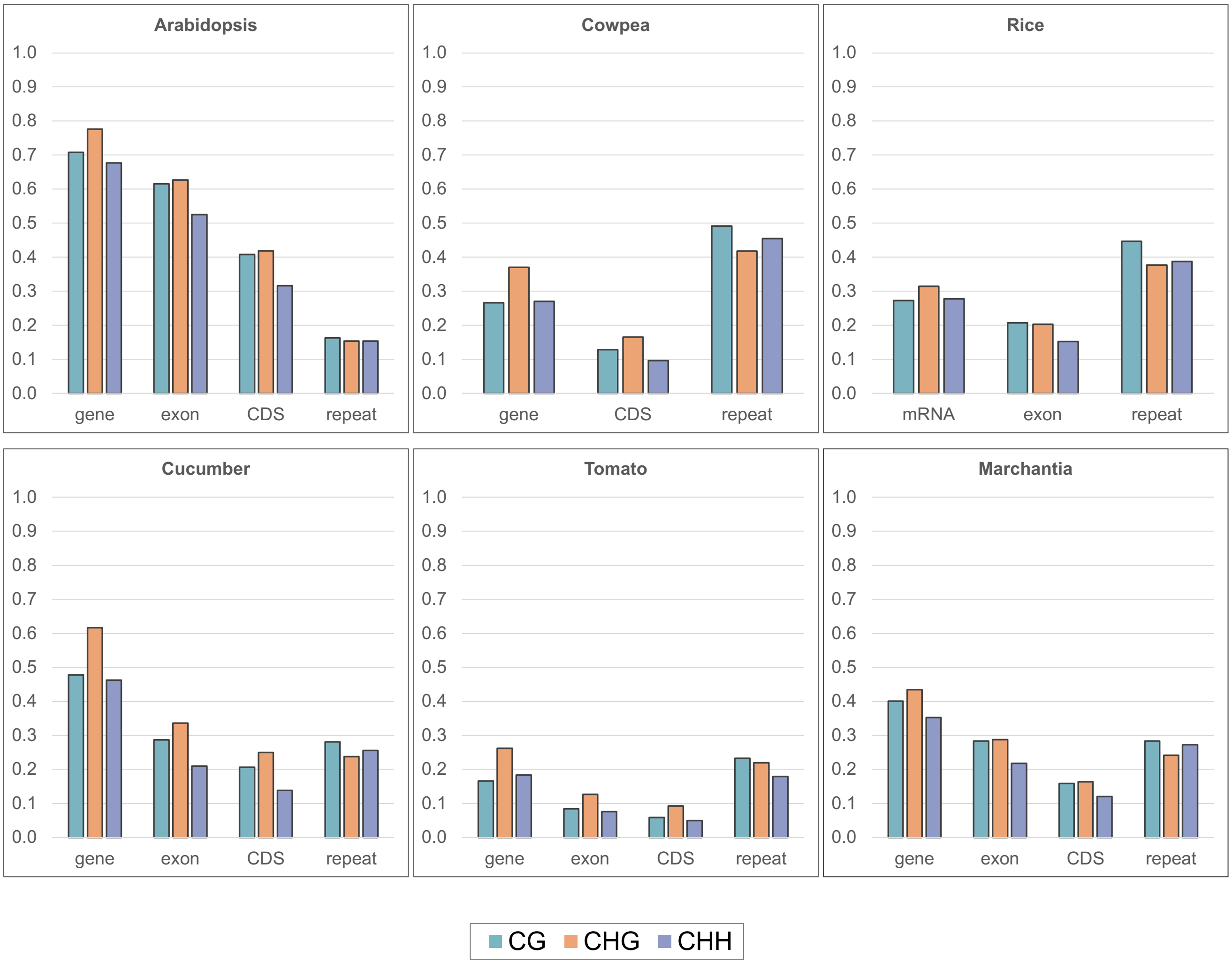

Supplementary Figure 19: Context-specific species-specific fraction of all cytosines covered by a functional annotation (see Supplemental Table 5 for the list) and repeats

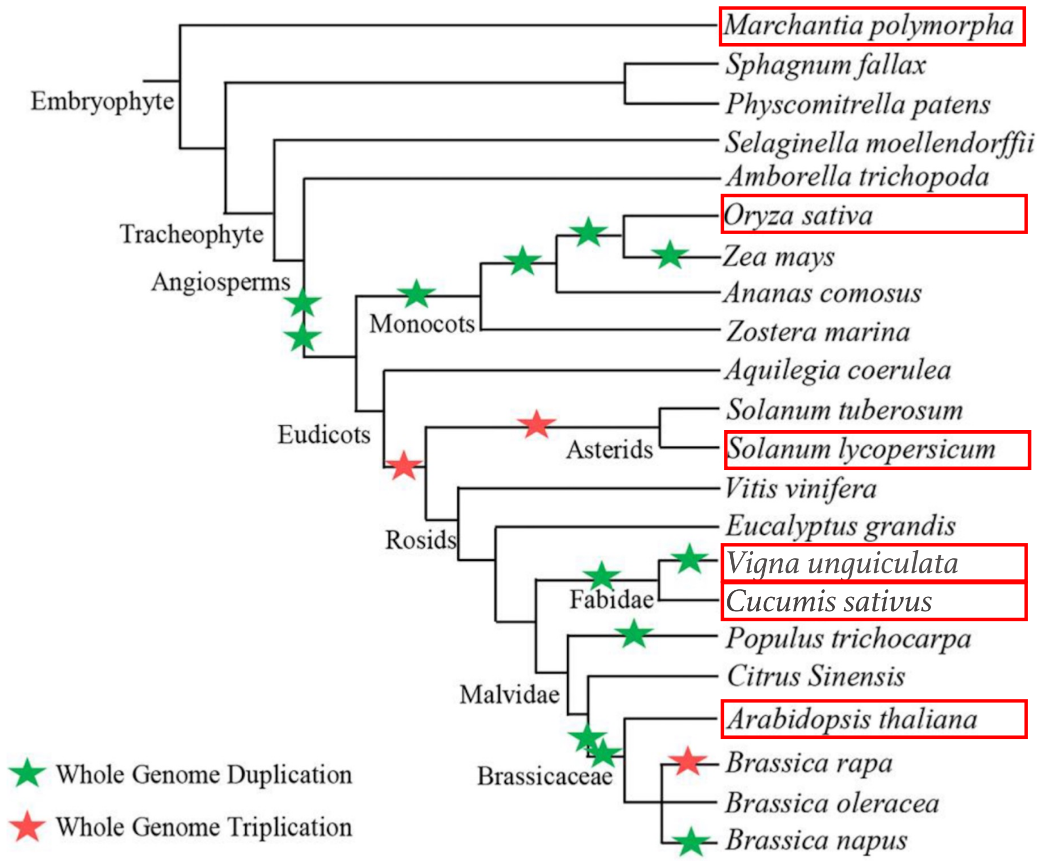

Supplementary Figure 20: A phylogenetic tree of land plants, decorated with whole-genome duplication events (adapted from `doi:10.3390/ijms20143591`); the six species included in this study are highlighted in red
